# Prevalence of cough throughout childhood: A cohort study

**DOI:** 10.1371/journal.pone.0177485

**Published:** 2017-05-24

**Authors:** Maja Jurca, Alban Ramette, Cristian M. Dogaru, Myrofora Goutaki, Ben D. Spycher, Philipp Latzin, Erol A. Gaillard, Claudia E. Kuehni

**Affiliations:** 1 Institute of Social and Preventive Medicine, University of Bern, Bern, Switzerland; 2 Faculty of Arts, Business and Applied Social Sciences, University Campus Suffolk, Ipswich, United Kingdom; 3 University Children’s Hospital Bern, University of Bern, Bern, Switzerland; 4 Division of Child Health, Department of Infection, Immunity and Inflammation, University of Leicester, Leicester, United Kingdom; University Children`s Hospital Zurich, SWITZERLAND

## Abstract

**Background:**

Cough in children is a common reason for medical consultations and affects quality of life. There are little population-based data on the epidemiology of recurrent cough in children and how this varies by age and sex, or between children with and without wheeze. We determined the prevalence of cough throughout childhood, comparing several standardised cough questions. We did this for the entire population and separately for girls and boys, and for children with and without wheeze.

**Methods:**

In a population-based prospective cohort from Leicestershire, UK, we assessed prevalence of cough with repeated questionnaires from early childhood to adolescence. We asked whether the child usually coughed more than other children, with or without colds, had night-time cough or cough triggered by various factors (triggers, related to increased breathing effort, allergic or food triggers). We calculated prevalence from age 1 to 18 years using generalised estimating equations for all children, and for children with and without wheeze.

**Results:**

Of 7670 children, 10% (95% CI 10–11%) coughed more than other children, 69% (69–70%) coughed usually with a cold, 34% to 55% age-dependently coughed without colds, and 25% (25–26%) had night-time cough. Prevalence of coughing more than peers, with colds, at night, and triggered by laughter varied little throughout childhood, while cough without colds and cough triggered by exercise, house dust or pollen became more frequent with age. Cough was more common in boys than in girls in the first decade of life, differences got smaller in early teens and reversed after the age of 14 years. All symptoms were more frequent in children with wheeze.

**Conclusions:**

Prevalence of cough in children varies with age, sex and with the questions used to assess it, suggesting that comparisons between studies are only valid for similar questions and age groups.

## Introduction

Cough is common during childhood. It leads to many medical consultations, affects quality of life and places a considerable burden on children, families and society [1]. Cough is a non-specific symptom of various diseases. Most often it is caused by acute respiratory infections, which can make children cough 5–8 times a year for 7–9 days each time [2]. In over 90% of children, this type of acute cough resolves by the third week of the infection [3]. Some children, however, have more frequent episodes (recurrent cough, non-acute cough), and questionnaire studies have used different questions to identify these children. This can indicate an underlying severe disease, such as cystic fibrosis, or primary ciliary dyskinesia. Studies from hospital patients with cough, who had a detailed diagnostic work-up, suggest that the most frequent underlying causes for recurrent cough are asthma, protracted bacterial bronchitis, chronic ear, nose and throat diseases, gastroesophageal reflux, and prolonged bronchial responsiveness after infections such as respiratory syncytial virus, rhinovirus or pertussis (post-infectious cough) [4]. Often, however, the underlying causes are not obvious (non-specific cough) [5].

Little is known about the epidemiology of childhood cough, assessed with different standardised questions. Most studies to date are cross-sectional and based on hospital patients [4,6]. Population-based studies are scarce [7–10]. Ongoing birth-cohort studies focus on wheeze, with little attention given to cough. Prevalence of cough has usually been reported for selected age groups only [11–13]. Studies used different questions on cough: most assessed dry cough at night, a key question of the International Study of Asthma and Allergies in Childhood (ISAAC) [11,14,15]. Others used two questions from the American Thoracic Society (ATS) questionnaire: cough occurring with colds and cough apart from colds [16,17]. Some studies asked if children coughed more than their peers [18,19]. To our knowledge, it has never been investigated how prevalence of cough varies throughout childhood in the general population, and whether results depend on how the questions on cough are formulated. Host and environmental factors change as a child grows up: the immune system and the lungs develop, the time spent outdoors and physical activity pattern change, lifestyle and exposure to allergens and toxins vary. All this might affect prevalence of cough.

In a large population-based cohort, we described prevalence of cough from age 1 to 18 years, using a variety of questions to assess cough. We did this for the entire study population, and separately for boys and girls. We also assessed cough prevalence in children with and without wheeze, because cough is a key symptom of childhood asthma and we were particularly interested in children who report cough without having asthma.

## Methods

### Study design and study population

This study used data from a large prospective population-based cohort in Leicestershire, UK [20]. The sampling frame was the Leicestershire Health Authority Child Health Database, a constantly updated database with perinatal, demographic and health-related information for all children resident in the Leicestershire Health Area. We extracted stratified random samples of children born between 1993 and 1997. Children of South Asian ethnic origin were oversampled. Perinatal and growth data were collected from birth records and health-visitor records during the first four years of life. Postal questionnaires on respiratory symptoms, diagnoses, treatments and environmental exposures were mailed to parents in 1998, 1999, 2001, 2003, 2006 and 2010. The response rate in 1998 was 78% (6808/8700), but lower afterwards [20].

The Leicestershire Health Authority Research Ethics Committee approved the study.

### The questionnaires

The questionnaire for the 1998 survey is a modified version of the questionnaire developed for the first Leicestershire cohort study, which recruited children born between 1985 and 1990 [20]. It contains questions from the ATS-childhood questionnaire [16], the key questions from ISAAC [14] and a few items from other validated questionnaires [20]. The same questionnaires were used in all subsequent surveys, with little variations to adapt the questions to growing age of children. Current wheeze (last 12 months) was assessed with the ISAAC key question [14], and wheeze was defined as ‘breathing that makes a high-pitched whistling or squeaking sound from the chest, not the throat’. We also assessed severity, whether the child ever had doctor-diagnosed asthma, and how many attacks of wheeze occurred in the past year. Repeatability of the questionnaire was good for questions on wheeze and moderate for questions on cough [21].

We asked several validated questions about cough, published in previous studies (Table 1): whether the child coughed more than other children [22]; whether he/she usually coughed with colds [16]; whether he/she coughed even without having a cold [16], and whether he/she had a dry cough at night, apart from cough associated with a cold or a chest infection [14]. We also asked about triggers for cough: triggers, related to increased breathing effort, including exercise or playing and laughing, excitement or crying; allergic triggers, such as house dust, pollen, or contact with pets; and cough triggered by food or drinks. Not all questions about cough were asked in all the surveys; the number of children who were asked a specific question is shown in supplementary S1 Table. In the first survey (1998) parents were asked whether their child had had frequent reflux during infancy. Parents completed the questionnaires until their child reached 13 years (surveys in 1998, 1999, 2001, 2003, 2006), children aged 14–17 (survey in 2010) completed the questionnaires themselves.

**Table 1:** Cough questions used in the Leicester Respiratory Cohort studies.

	Source of the question
**Coughing more than peers**	**Robertson et al**. Prevalence of asthma in Melbourne schoolchildren: changes over 26 years. *BMJ* 1991 [22]
*Do you think that your child coughs more than other children*? □ yes □ no	
**Cough with colds**	**Ferris BG**. Epidemiology Standardization Project (American Thoracic Society). *Am Rev Respir Dis* 1978 [16]
*Does your child usually have a cough with colds*? □ yes □ no	
**Cough apart from colds**	**Ferris BG**. Epidemiology Standardization Project (American Thoracic Society). *Am Rev Respir Dis* 1978 [16]
*Does your child have a cough even without having a cold*? □ no, never □ yes, sometimes □ yes, always	
**Night cough**	**Asher et al**. International Study of Asthma and Allergies in Childhood (ISAAC): rationale and methods. *Eur Respir J* 1995 [14]
*In the last 12 months*, *has your child had a dry cough at night*, *apart from a cough associated with a cold or a chest infection*? □ yes □ no	
**Cough triggers**	*Newly developed*. **Strippoli**. A parent-completed respiratory questionnaire for 1-year-old children: repeatability. *Arch Dis Child* 2007 [21]
*In the last 12 months*, *did the following things cause your child to cough*? □ Exercise (playing, running) □ Laughing, crying □ House dust □ Pollen (grass, hay, trees, flowers) □ Contact with pets or other animals □ Food or drinks	

### Statistical analyses

This study included data of all children who participated in at least one survey (7670/8700, 88%). Due to the range in birth years (1993–7), children were aged 1–4 years at the first survey in 1998 and 13–17 in 2010. Data were analysed by age groups, rather than by the calendar year of the surveys (S1 Fig). We made seven age groups: 1-year-olds, 2-year-olds, 3-4-year-olds, 5–6, 7–9, 10–13, and 14-17-year-olds, choosing narrow age intervals in early childhood, because changes in respiratory physiology and lifestyle occur faster during infancy and preschool years than later on. Children were included only once in each age group, but could have contributed observations from different surveys to several age groups. We had data on 4102 1-year-olds, 3163 2-year-olds, 4071 3-4-year-olds, 4031 5-6-year-olds, 3244 7-9-year-olds, 2204 10-13-year-olds, and 2025 14-17-year-olds (S1 Table).

We estimated prevalence of cough for each age group and for the 10 cough questions. We did this first for all children, to obtain prevalence estimates for the general population. Then, we computed prevalence estimates separately for boys and girls. We performed likelihood ratio tests for the difference in cough prevalence between boys and girls. We investigated further, how prevalence of cough changes with age using logistic generalised estimating equation (GEE) models with an exchangeable working correlation structure to account for repeated measurements of cough [23]. Odds ratios (ORs), comparing prevalence of cough in different age groups to the baseline age group of 1-year-olds, were estimated with 95% CI using robust standard errors to account for the repeated observations from each child. We compared prevalence for the entire population, and separately for children with and without wheeze. In a sensitivity analysis, we divided children with wheeze based on severity of wheeze (doctor-diagnosed asthma, frequent episodes of wheeze attacks) and compared prevalence of cough in the subgroup of participants with severe wheeze to the one in children with less severe wheeze.

Finally, we performed a sensitivity analysis where we accounted for potential attrition bias using inverse probability weighting. Additional information on this method is available in the online supplement (S1 Text).

We used STATA software to prepare the dataset and conduct all analyses (Stata 14.1; Stata Corporation, Austin, TX, USA).

## Results

In 1998, most children (59%) were aged 1 year, 52% were boys, and 27% were of South Asian ethnic origin (Table 2). Prevalence of current wheeze was 34% in 1-year-olds, 23% in 2-year-olds, 19% in 3-4-year-olds, and about 15% thereafter. Prevalence of doctor-diagnosed asthma (ever in life) increased from 12% at age 1 to 23% at age 14–17 years (S2 Table). Among children with current wheeze, prevalence of doctor-diagnosed asthma increased from 33 to 64% throughout childhood, among non-wheezers from 3 to 16% (S3 Table).

**Table 2:** Characteristics of the study population in 1998 (N = 6808).

		n	%
**Demographic factors**			
Age	1 year	3983	59
	2 years	950	14
	3 years	871	13
	4 years	886	13
Sex	Male	3547	52
	Female	3261	48
Ethnicity	White	4986	73
	South Asian	1822	27
**Perinatal and early life factors**			
Low birth weight (<2500g)		473	7
Gestational age <37 weeks		418	6
Breastfed		4035	60
**Environmental exposures**			
Nursery care		2985	44
Mother smoking (postnatally)		1349	20
Other person smoking in household		1744	26
**Socioeconomic factors**			
Higher parental education^#^		2851	42
More deprived (Townsend deprivation index)^¶^		1332	20
**Parental history of atopic diseases**			
Hay fever (mother or father)		2954	43
Wheeze or asthma (mother or father)		1955	29
**Symptoms**			
Wheeze		2025	30
Asthma (doctor diagnosed) ^+^		550	17
Gastroesophageal reflux (frequent posseting)		1044	15
Frequent colds^§^		1194	18
Coughing more than peers^+^		328	10
Cough with colds		4849	71
Cough without colds		2150	32
Night cough		1809	27
*Cough triggered by*:			
Exercise/play		877	14
Laughter/crying^+^		756	22
House dust^+^		105	3
Pollen^f^		-	-
Pets		118	2
Food/drinks		700	11

^#^: age at the end of education is >16 years;

^¶^: Townsend Deprivation Index: more affluent [-6.222, -2.635], affluent [-2.615, -0.707], average [-0.705, 1.859], deprived [1.861, 5.147], more deprived [5.160, 11.072];

^+^: the question was asked only in a subcohort;

^§^: ≥7 colds in the last 12 months;

^f^: not asked in the 1998 survey.

### Prevalence of cough throughout childhood; entire study population

**Fig 1:**
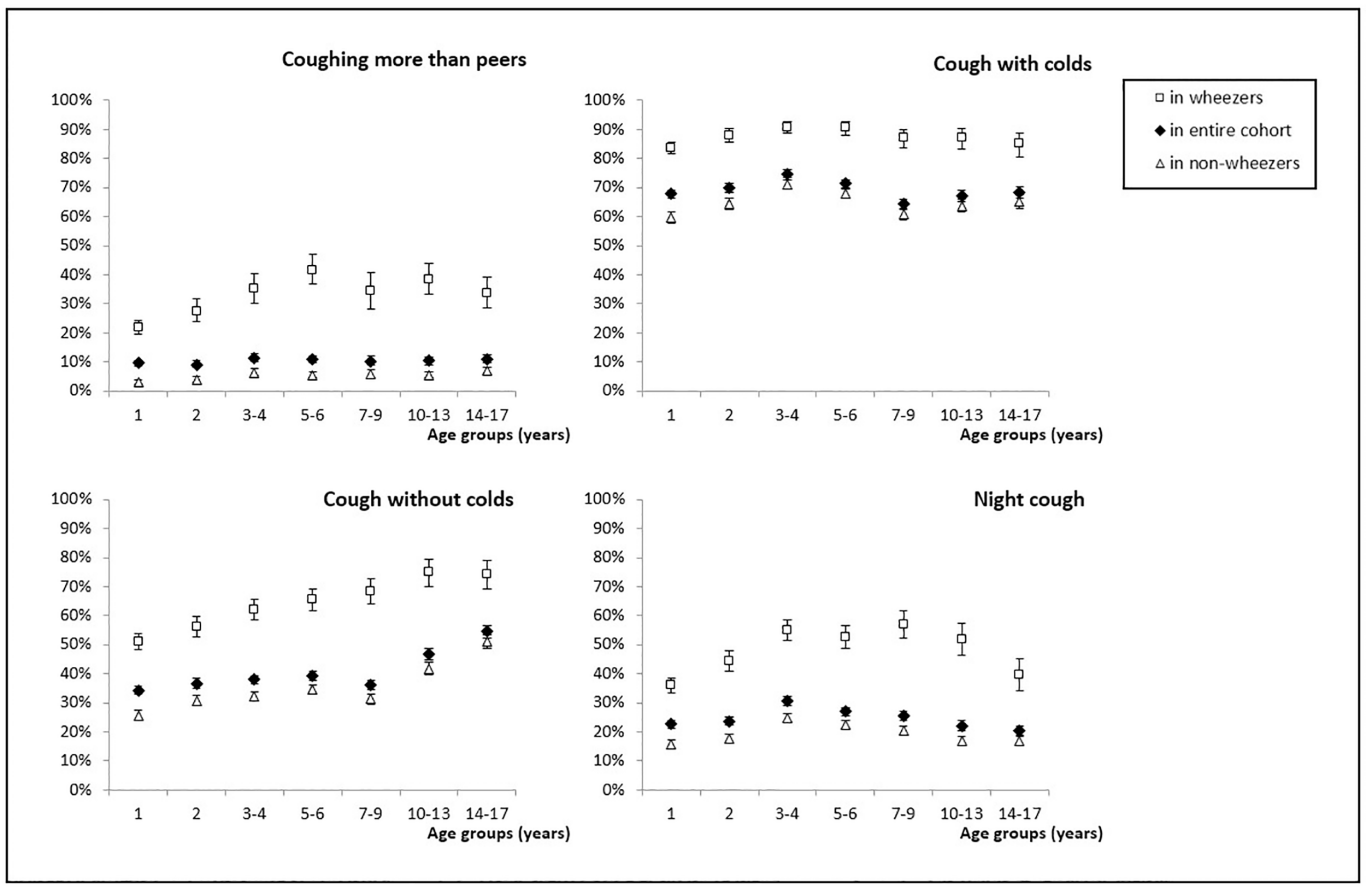
Prevalence of coughing more than peers, cough with colds, cough apart from colds and night cough in the entire cohort, and among children with wheeze and non-wheezers, for different age groups. Prevalence (%) with 95% confidence intervals.

**Fig 2:**
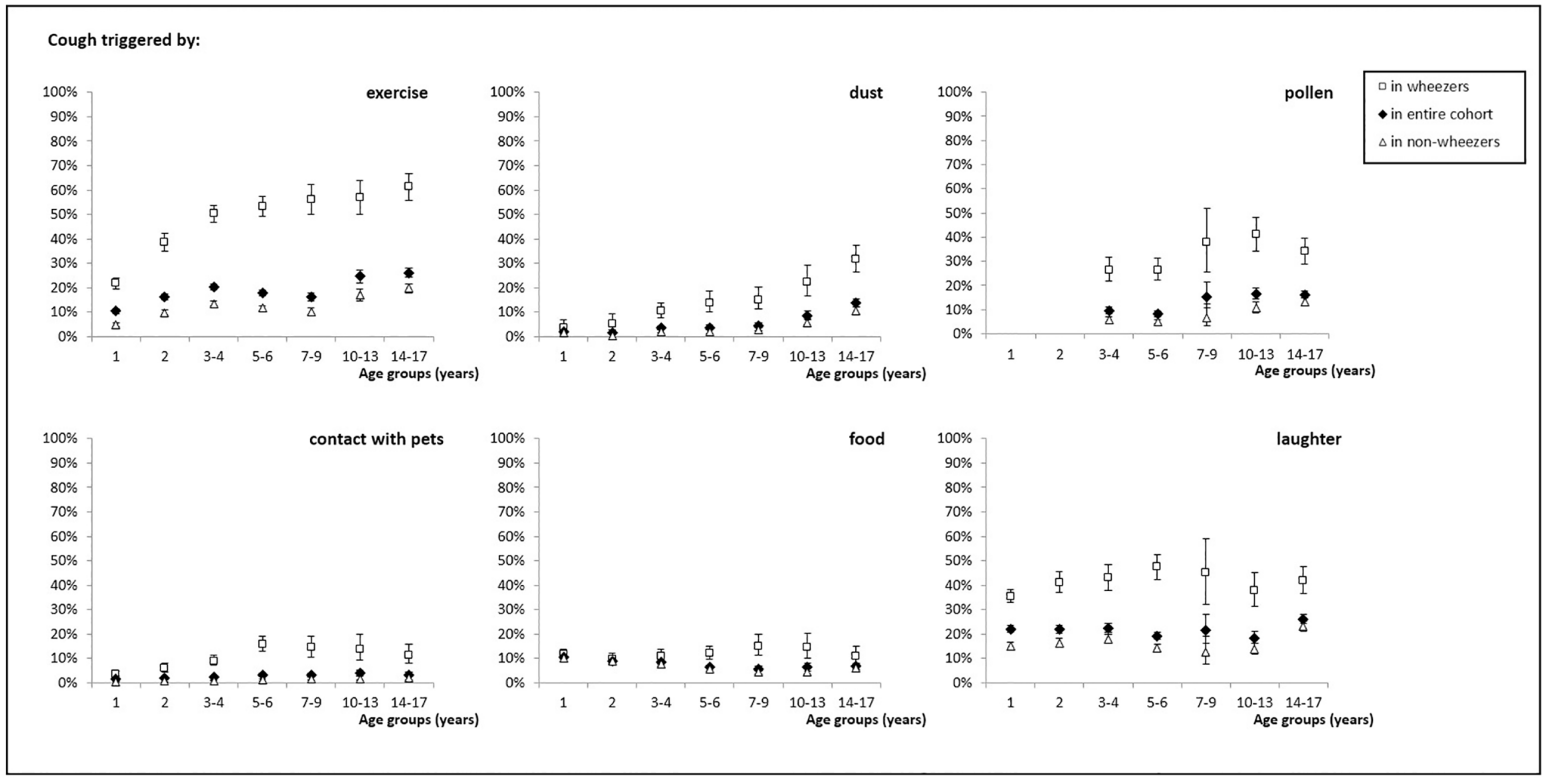
Prevalence of cough triggered by exercise, house dust, pollen, pets, food and laughter or crying in the entire cohort, and among children with wheeze and non-wheezers, for different age groups. Prevalence (%) with 95% confidence intervals.

Prevalence of cough varied significantly, depending on the questions used to assess it (Figs 1 and 2, Table 3; S4 Table). Ten percent (95%CI 10–11) of parents said that their child coughed more than peers, with little differences between age groups (Fig 1). Sixty-nine percent of children (69–70%) were reported to cough usually with colds; there were only small differences between age groups. In contrast, the prevalence of cough without colds increased with age from 34% in 1-year-olds to 55% in children aged 14–17. Night cough was reported for 25% (25–26%) of children. It increased slightly from 23% in 1-year-olds to 31% in 3-4-year-olds, and then decreased again to 20% at age 14–17.

**Table 3:** Prevalence of cough in different age groups in the entire cohort, and separately for children with and without wheeze.

**Age group**	**1-year-olds** **(N = 4102)**		**2-year-olds** **(N = 3163)**		**3-4-year-olds** **(N = 4071)**		**5-6-year-olds** **(N = 4031)**		**7-9-year-olds** **(N = 3244)**		**10-13-year-olds** **(N = 2204)**		**14-17-year-olds** **(N = 2025)**	
*Entire cohort*	n	%(CI)	n	%(CI)	n	%(CI)	n	%(CI)	n	%(CI)	n	%(CI)	n	%(CI)
Coughing more^#^	313	10(9–11)	213	9(8–10)	217	11(10–13)	257	11(10–12)	147	10(9–12)	227	10(9–12)	221	11(10–12)
Cough with colds	2790	68(67–69)	2205	70(68–71)	3043	75(73–76)	2880	71(70–73)	2086	64(63–66)	1482	67(65–69)	1382	68(66–70)
Cough without colds	1403	34(33–36)	1159	37(35–38)	1544	38(36–39)	1579	39(38–41)	1175	36(35–38)	1031	47(45–49)	1104	55(52–57)
Night cough^¶^	931	23(21–24)	750	24(22–25)	1245	31(29–32)	1088	27(26–28)	824	25(24–27)	486	22(20–24)	412	20(19–22)
*Cough triggers*^¶^:														
Exercise/play^#^	417	10(9–11)	494	16(15–17)	795	20(19–22)	695	18(17–19)	307	16(15–18)	244	25(22–27)	529	26(24–28)
Laughter/crying^#^	728	22(21–24)	514	22(20–24)	427	22(21–24)	452	19(18–21)	41	22(16–28)	182	18(16–21)	529	26(24–28)
House dust^#^	18	2(1–3)	14	2(1–3)	80	4(3–5)	63	4(3–5)	86	5(4–6)	82	9(7–11)	264	14(12–15)
Pollen^#^	-	-	-	-	176	10(8–11)	191	8(7–9)	29	15(11–21)	163	17(14–19)	328	16(15–18)
Pets^#^	61	2(1–2)	55	2(1–2)	95	2(2–3)	134	3(3–4)	64	3(3–4)	37	4(3–5)	65	3(3–4)
Food/drinks^#^	424	11(10–12)	276	9(8–10)	333	8(8–9)	263	7(6–7)	115	6(5–7)	60	6(5–8)	130	7(6–8)
	**1-year-olds** **(N = 1409)**		**2-year-olds** **(N = 726)**		**3-4-year-olds** **(N = 761)**		**5-6-year-olds** **(N = 606)**		**7-9-year-olds** **(N = 434)**		**10-13-year-olds** **(N = 331)**		**14-17-year-olds** **(N = 309)**	
*Wheezers*	n	%(CI)	n	%(CI)	n	%(CI)	n	%(CI)	n	%(CI)	n	%(CI)	n	%(CI)
Coughing more^#^	249	22(19–24)	142	27(24–31)	118	35(30–40)	147	42(37–47)	77	34(28–41)	127	38(33–44)	104	34(29–39)
Cough with colds	1184	84(82–86)	639	88(86–90)	692	91(89–93)	549	91(88–93)	379	87(84–90)	289	87(83–91)	263	85(81–89)
Cough without colds	718	51(48–54)	409	56(53–60)	473	62(59–66)	398	66(62–69)	297	68(64–73)	248	75(70–79)	230	74(69–79)
Night cough^¶^	506	36(33–38)	320	44(41–48)	420	55(52–59)	320	53(49–57)	247	57(52–62)	172	52(47–57)	123	40(34–45)
*Cough triggers*^¶^:														
Exercise/play^#^	296	22(20–24)	267	39(35–42)	364	50(47–54)	310	53(49–57)	140	56(50–62)	109	57(50–64)	190	61(56–67)
Laughter/crying^#^	411	36(33–39)	214	41(37–46)	145	43(38–49)	167	47(42–53)	24	45(32–59)	72	38(31–45)	130	42(36–48)
House dust^#^	10	4(2–7)	11	5(3–9)	44	10(8–14)	35	14(10–19)	37	15(11–20)	40	22(17–29)	91	32(27–37)
Pollen^#^	-	-	-	-	87	27(22–32)	93	26(22–31)	20	38(25–52)	78	41(34–48)	105	34(29–40)
Pets^#^	47	4(3–5)	41	6(4–8)	66	9(7–11)	93	16(13–19)	38	14(11–19)	24	14(9–20)	34	12(8–16)
Food/drinks^#^	158	12(10–14)	70	10(8–12)	82	11(9–14)	70	12(10–15)	40	15(11–20)	26	14(10–20)	31	11(8–15)
	**1-year-olds** **(N = 2693)**		**2-year-olds** **(N = 2437)**		**3-4-year-olds** **(N = 3310)**		**5-6-year-olds** **(N = 3425)**		**7-9-year-olds** **(N = 2810)**		**10-13-year-olds** **(N = 1873)**		**14-17-year-olds** **(N = 1716)**	
*Non-wheezers*	n	%(CI)	n	%(CI)	n	%(CI)	n	%(CI)	n	%(CI)	n	%(CI)	n	%(CI)
Coughing more^#^	64	3(2–4)	71	4(3–5)	99	6(5–8)	110	6(5–7)	70	6(5–7)	100	5(4–7)	117	7(6–8)
Cough with colds	1606	60(58–62)	1566	64(62–66)	2351	71(69–73)	2331	68(66–70)	1707	61(59–63)	1193	64(61–66)	1119	65(63–67)
Cough without colds	685	25(24–27)	750	31(29–33)	1071	32(31–34)	1181	34(33–36)	878	31(30–33)	783	42(40–44)	874	51(49–53)
Night cough^¶^	425	16(14–17)	430	18(16–19)	825	25(23–26)	768	22(21–24)	577	21(19–22)	314	17(15–19)	289	17(15–19)
*Cough triggers*^¶^:														
Exercise/play^#^	121	5(4–5)	227	10(8–11)	431	14(12–15)	385	12(11–13)	167	10(9–12)	135	17(14–20)	339	20(18–22)
Laughter/crying^#^	317	15(13–17)	300	16(15–18)	282	18(16–20)	285	14(13–16)	17	13(8–19)	110	14(12–16)	399	23(21–25)
House dust^#^	8	1(1–3)	3	0.5(0.2–1.5)	36	2(1–3)	28	2(1–3)	49	3(2–4)	42	5(4–7)	173	11(9–12)
Pollen^#^	-	-	-	-	89	6(5–7)	98	5(4–6)	9	7(3–12)	85	11(9–13)	223	13(11–15)
Pets^#^	14	0.5(0.3–0.9)	14	0.6(0.4–1)	29	0.9(0.6–1.3)	41	1(1–2)	26	1.5(1–2)	13	2(1–3)	31	2(1–3)
Food/drinks^#^	266	10(9–11)	206	9(8–10)	251	8(7–9)	193	6(5–7)	75	4(3–5)	34	4(3–6)	99	6(5–7)

CI: confidence interval;

^#^: only asked in part of the cohort;

^¶^: symptoms occurring in the past 12 months.

Replies to questions on triggers for cough varied strongly with age (Fig 2, Table 3; S4 Table). Ten percent of 1-year-olds were reported to cough after exercise or playing; this increased to 26% in the oldest age group. About a quarter of children in all age groups (range 18–26%) were reported to cough after laughing or crying. The prevalence of cough triggered by house dust increased from 2% in the youngest children to 14% in the oldest. Cough triggered by pollen increased from 10% in 3-4-year-olds to 15% in 7-9-year-olds, with little change thereafter. Cough triggered by pets was rare (2–4% of children) in all age groups. The proportion of families owning a pet ranged between 41 and 51% across childhood. The prevalence of cough triggered by food or drinks decreased slowly from 11% in the youngest age group to 6% in 7-9-year-olds, with little change thereafter. There was a significant association between reports of cough triggered by food and frequent reflux in infancy; 18% of 1-4-year-old children with frequent reflux were reported to have cough triggered by food compared to 6% of children without (p<0.001) (S5 Table). Children whose cough was triggered by food, had around twice as high prevalence of night cough, compared to children without cough triggered by food throughout childhood (p<0.001).

### Sex-stratified prevalence of cough throughout childhood

We found small differences in prevalence of cough between boys and girls (S6 Table, S2 and S3 Figs). Cough was slightly more prevalent in boys than in girls until the completed age of six for the following questions: coughing more than peers, exercise-induced cough and cough, triggered by pollen. Similar differences were found for cough without colds, at night and cough, triggered by pets or laughter for younger children (toddlers). The reverse was found in adolescents; girls reported slightly more cough without colds, at night and triggered by food or laughter.

### Prevalence of cough throughout childhood; in children with and without wheeze

All types of cough were reported much more often for children with current wheeze than for children without (Figs 1 and 2, Table 3; S4 Table). Depending on the questions, prevalences were 2–10 times higher in children with wheeze compared to non-wheezers. However, the age-related changes in prevalences were similar in both groups, although on a lower level for non-wheezers.

Among children with wheeze, 22% to 42% were reported to cough more than peers; this was 3–7% among non-wheezers (Fig 1, Table 3; S4 Table). Eighty-seven percent (CI 86–88%) of wheezing children and 65% (64–66%) of non-wheezers usually coughed when they had a cold. Prevalence of cough without colds was reported for 51% of infants with wheeze and 25% of infants without; and increased in prevalence until age 14–17 years in both groups. Night cough was reported for 46% (45–48%) of children with wheeze, and 20% (19–21%) of children without.

Prevalence of cough triggered by exercise increased from 22% at age 1 to 61% at age 14–17 in children with wheeze, and from 5 to 20% in children without (Fig 2, Table 3; S4 Table). Thirty-six to forty-seven percent of children with wheeze and 15–23% of those without were reported to cough after laughing/crying. Cough triggered by house dust increased from 4 to 32% in children with wheeze and from 1 to 11% in the others. Cough triggered by pollen ranged between 26% and 41% in wheezers, depending on the age group, and from 5 to 13% in non-wheezers. Between 4% and 16% of children with wheeze were reported to cough after contact with pets, but only 0.5–2% of those without. Cough triggered by food/drinks was the only symptom that was reported with equal frequency (9–12% in children with and without wheeze at ages 1 and 2 years). From age 3–4 years onwards, prevalence decreased in non-wheezers and increased in wheezers, resulting in a widening gap between the groups. Cough triggered by food was significantly associated with reflux both in children with and without current wheeze: among 1-4-year-olds with wheeze 19% of those with reported reflux but only 8% of those without reflux reported cough triggered by food or drinks (p<0.001); the same was true for 1-4-year-olds without wheeze, where 17% of those with and 5% of those without reflux had cough triggered by food or drinks (p<0.001). Also sex-differences in prevalence of cough were comparable in wheezers and non-wheezers (S6 Table).

Children with current wheeze who had also a doctor’s diagnosis of asthma, and those with frequent wheeze (more than four attacks in the past year) had a higher prevalence of cough than those without an asthma diagnosis, or infrequent wheeze (S7 Table). For instance, among 5-6-year-old children with frequent wheeze, 63% were reported to cough more than peers and 67% had night-time cough. Among 10-13-year-olds with frequent wheeze, 78% had cough without colds and 76% exercise-induced cough. Among children with current wheeze and asthma diagnosis, 81% of 10-13-year-olds coughed without colds.

The weighted results from the sensitivity analyses were very similar to the unweighted results (online S8 Table); therefore, we presented unweighted results in the printed paper for simplicity.

## Discussion

This study describes cough prevalence systematically from infancy through adolescence in an unselected childhood cohort, using a series of questions to assess cough. We found that: 1) prevalence estimates of cough varied widely depending on the questions assessing it; 2) there were distinct age-related changes in prevalence, which differed between cough questions; and 3) all types of cough were more common in children with wheeze, but age-related prevalence patterns were similar in children with and without wheeze.

To our knowledge, ours is the first study that describes prevalence of cough, assessed by multiple questions, in a population-based sample of children covering all age groups from infancy through adolescence and with prevalences stratified by sex and by presence of wheeze. Its strengths include the large size, the fact that it is based on an unselected sample of children from the general population, that it included children of all ages (1–18 years), and used many questions to inquire about cough. Surveys were conducted every 2–3 years, always in late spring (May), used a consistent methodology, and repeated the same questions. The repeatability of the cough questions was assessed for 1-year-olds by resending the questionnaires after 3 months (August). The kappa coefficients were 0.57 for coughing more than peers, 0.46 and 0.54 for coughing with and without colds, 0.39 for night cough, and 0.53, 0.57, 0.61, and 0.49 for cough triggered by exercise, pets, food and laughter (moderate repeatability) [21]. This reflects seasonal variation in cough prevalence. It should not have biased the comparison between age groups, since all surveys had been conducted in the same season. Because there was attrition in the cohort, and we did not know if responders might differ from non-responders in cough prevalence, we performed a sensitivity analysis with appropriate weights, adjusting for potential non-response bias. These prevalence estimates were very similar to estimates in the original unweighted analysis, which suggests our results are representative of the general population. A weakness of the study is that we had not assessed measures of severity of cough, for instance, duration of episodes or sputum production. Questions on the duration of cough episodes were only included in the 2010 questionnaire, but not in the others, and could thus not be described for all age groups. This study also did not describe specific diseases leading to cough, such as whooping cough, and did not clearly differentiate between wet and dry cough. Parental or self-reporting of respiratory symptoms is a pragmatic tool in large epidemiological studies. However, it has been shown that parental reporting of cough is not very accurate compared to objective measurements [24,25]. We cannot exclude a differential recall bias, in that parents of children with wheeze might be alert to hearing and reporting night-time cough, because this is a question that doctors ask them at follow-up visits. Additionally, though symptoms from age 1–13 were parent-reported, symptoms in 14-17-year-olds were self-reported. We therefore cannot say whether changes in prevalence between the age groups 10–13 and 14–17 reflect a biological change, related to age, or a reporting difference between children and their parents [26].

Surprisingly few studies have assessed prevalence of cough in the general population (Table 4). Most studies asked only one or two questions about cough, usually the ISAAC core question on night cough, and investigated one or two age groups. Many studies described night cough in 6-7-year-olds and 13-14-year-olds as part of the ISAAC international surveys [11]. Pearce et al. compared the prevalence of asthma symptoms worldwide in almost 500,000 children [11]. Night-cough prevalence showed significant geographical variability in parallel with wheeze, with higher prevalence in English-language countries and Western Europe, and lower prevalence in Eastern Europe. Results from the UK were similar to ours: 27% for 6–7 and 19% for 13-14-year-olds. Two other British studies found slightly higher prevalence of night cough than our study [12,15], but one was conducted in patients from a deprived suburb area, registered in a general practice [12]. Robertson et al. examined night cough in almost 11,000 children from Australia, aged 7 (cough prevalence 28%), 12 (19%) and 15 years (16%), with similar findings to ours [22,27]. Night-cough prevalence in France [13], Germany [28,29], Switzerland [27,30], the Netherlands [31,32], Eastern and Northern Europe [33,34] was lower, and in Chile [27] higher, than what we found. This parallels known geographical differences for prevalence of wheeze and asthma [11].

**Table 4:** International comparison of age-specific prevalence of cough, assessed by different questions.

Age groups (years)			1	2	3–4	5–6	7–9	10–13	14–17
Study	Country	N							
*Prevalence of night cough*			% (95% CI)	% (95% CI)	% (95% CI)	% (95% CI)	% (95% CI)	% (95% CI)	% (95% CI)
Jurca et al.2017	UK	7670	23 (21–24)	24 (22–25)	31 (29–32)	27 (26–28)	25 (24–27)	22 (20–24)	20 (19–22)
Pearce et al.2007 [11]	UK (Sunderland)	1843^#^,2193^¶^				27^#^			19^¶^
	Austria	6876^#^,1439^¶^				12^#^			19^¶^
	Germany	3830^#^,4132^¶^				16^#^			23^¶^
	Sweden	2089^#^,2679^¶^				12^#^			12^¶^
	Poland	4496^#^,4420^¶^				25^#^			22^¶^
Linehan et al.2005 [12]	UK	1869^+^	31	29	40				
Burr et al.1999 [15]	UK	25393						45^§^	
Robertson et al.1993 [27]	Australia	10981					28^f^	19^##^	16^g^
	Switzerland	4464					17^f^	11^##^	8^g^
	Chile	11183					37^f^	35^##^	28^g^
Ranciere et al.2013 [13]	France	1869	14	14	18^###^				
Morgenstern et al.2007 [28]	Germany	3577	7	14					
Gehring et al.2002 [29]	Germany	1756	7	14					
Sennhauser et al.1995 [30]	Switzerland	4353					17^f^	11^##^	9^g^
Gehring et al.2011 [31]	Netherlands	3861	18	16	21	23**	14***		
Brauer et al.2007 [32]	Netherlands	3537			22				
Leonardi et al.2002 [33]	Eastern Europe	21743					12^h^		
Timonen et al.1995 [34]	Eastern Finland	2564					12^h^		
*Prevalence of cough without colds*									
Jurca et al.2017	UK	7670	34 (33–36)	37 (35–38)	38 (36–39)	39 (38–41)	36 (35–38)	47 (45–49)	55 (52–57)
Burr et al.1999 [15]	UK	25393						29^§^	
Luyt et al.1993 [17]	UK	1422	17	19	24	28			

N: number of study participants; CI: confidence interval; UK: United Kingdom;

^#^: in 6-7-year-olds;

^¶^: in 13-14-year-olds;

^+^: children registered at general practitioner;

^§^: in 12-14-year-olds;

^f^: in 7-year-olds;

^##^: in 12-year-olds;

^g^: in 15-year-olds;

^###^: 17% in 3-year-olds, 19% in 4-year-olds;

**: in 5-year-olds;

***: in 8-year-olds;

^h^: in 7-11-year-olds.

The other cough questions have hardly been studied in unselected samples of children so far. Two older surveys, the first Leicester Respiratory Cohort study conducted in children born 1985–1990 [17], and a study by Burr et al. [15], reported lower prevalence of cough without colds than we found in the second Leicester cohort. This mirrors secular changes in prevalence of wheeze in the Leicestershire region [35].

The prevalence of cough varied minimally by sex, but in the same direction as the variations described for wheeze [36], which is usually more frequent in boys during preschool and early school years, but more frequent in girls from about 16 years onwards [37]. In our study, we found that prevalence of cough was higher in boys until the age of about 13 but higher in girls thereafter—as also found in other studies for selected questions [15,37]. The gender difference was most pronounced for types of cough, which have been reported to be related to asthma (cough more than peers, cough without colds, cough at night, cough triggered by exercise or certain aeroallergens). Hence, some of the underlying mechanisms for gender differences might be similar for cough and wheeze. Bronchial hyperresponsiveness in childhood is more common and more severe among boys, but girls catch up during adolescence [36]. Second, sex-based differences in the anatomy of the respiratory system have been described as dysanapsis—boys have narrower airways related to the lung volume, caused by disproportionate growth of airways and lung parenchyma [38]. The reverse is seen in adults where airway diameters are about 17% larger in men [39]. A third mechanism could be age-related changes in cough sensitivity: some studies in adults showed higher cough sensitivity in women [40–42], while Chang et al. found no sex differences in cough receptor sensitivity in young children [43,44].

Only few studies measured cough prevalence separately in children with and without wheeze. We confirmed that the cough prevalence is much higher in children who wheeze than in non-wheezers. Brooke et al. has previously shown that night-cough prevalence was 39% in 4-8-year-olds with wheeze, and 19% in those without [25]. Von Mutius et al. assessed exercise-induced cough with a slightly different question, and reported that 15% of 9-11-year-olds overall, and 51% of those with asthma, had exercise-induced cough. Results for children without wheeze were not given [45]. Night-time cough and cough without colds are thought to be key symptoms of childhood asthma and are usually asked in consultations. Surprisingly, only a quarter of all children, and only 46% of children who have wheeze, complained about night cough. The proportion increased in children with frequent wheeze, or those with doctor-diagnosed asthma, but was not higher than 67% for night cough or than 81% for cough apart from colds. This could reflect differences in importance and occurrence of cough between different asthma phenotypes, as found in a previous study that considered chronic cough in a multivariate model to define asthma phenotypes [46].

Overall, prevalence of cough varies throughout childhood in distinctive age-related patterns, and results differ depending on which exact question is used to assess cough. About 10% of parents, independent of their child’s age, think that their child coughs more than others; about 30% of parents whose children wheeze, but only about 5% of parents whose children do not wheeze. This suggests that the burden of recurrent cough in children without wheeze is relatively low. Unexpectedly, only about 70% of children report coughing with colds. This could signal differences in cough receptor sensitivity, as children may have variable thresholds for reflex initiation [47]. Cough without colds gets more common with age. Reasons might include cough caused by smoking, or because of undiagnosed asthma [48]. Asthma is often underdiagnosed in adolescents, and cough is the most common symptom among adolescents with undiagnosed asthma [49].

Prevalence of cough triggered by different factors varied strongly with age. Our data do not allow making any deduction on the underlying causes of cough. However, based on other literature, we can make some speculations, which need to be verified by further research. Cough triggered by exercise was more frequent in teenagers than in younger children, probably because teenagers are more physically active or perform more strenuous activities. Cough triggered by laughter or crying remained comparatively constant throughout childhood. Its pathophysiological mechanisms are probably similar to exercise-induced cough (increased breathing effort, dry airways, consequently cough) [50]. It has been suggested that these children have low threshold mechanosensors, which render even mild mechanical triggers irritating, possibly as part of cough hypersensitivity syndrome [47]. Prevalence of coughing due to allergic triggers (house dust, pollen and pet dander) increased with age. We know that allergic sensitisation to allergens increases with age and some monosensitised children become polysensitised [51]. We found similar prevalence of food-induced cough in small children, whether they had current wheeze or not. This suggests that the causes of cough triggered by food might vary by age. In infants, the main cause is likely to be gastroesophageal reflux [52]. In fact, cough triggered by food was reported more often in young children with reflux (17–20%) compared to those without (5–7%). In toddlers, food allergies might become more important, as reflected in our study; children with wheeze had more food-induced cough and the cough prevalence diverged between the presence and absence of wheeze in older children. There was more night-time cough among children who reported cough triggered by food compared to children without, suggesting that a proportion of these infants aspirate when they are in supine position at night.

In summary, our study describes for the first time prevalence of cough throughout the whole childhood, with a standardised approach and a wide variety of questions to assess cough. We found large differences between different cough questions, implying that the choice of questions relating to cough and their phrasing are essential, and comparisons between studies are only valid for similar questions and age groups. Future studies should select the questions on cough carefully, depending on the exact aim of the study and age of participants. We found differences between girls and boys, and differences in age-related cough patterns, with older children more commonly reporting exercise-induced cough, and cough in conjunction with aeroallergen sensitisation. The age-related patterns are likely explained by physiological changes in the growing child and changing exposures to environmental factors.

## Supporting information

S1 Text Methods supporting information. Sensitivity statistical analysis.
(DOCX)

S1 Fig Age of the children of the Leicester Respiratory Cohorts participating at the respective surveys. The bands on the x-axis show the seven age groups used for the analysis (1, 2, 3–4, 5–6, 7–9, 10–13 and 14-17-year-olds). The y-axis shows the years when surveys were conducted. In 1999, only those who responded in 1998 and were born in years 1996–7 (aged 1 year at the first survey) were addressed.
(TIFF)

S2 Fig Prevalence of cough in entire cohort, stratified by sex. (TIF)

S3 Fig Prevalence of cough triggered by different factors in entire cohort, stratified by sex. (JPG)

S1 Table Total number of observations for questions on cough and information on missing data. Number of children who were asked about certain aspects of cough in different surveys of Leicester Respiratory Cohorts and reported results with missing data, stratified by age group.
(DOCX)

S2 Table Prevalence of wheeze and doctor-diagnosed asthma in different age groups. (DOCX)

S3 Table Prevalence of doctor-diagnosed asthma (ever) in different age groups, stratified by current wheeze. (DOCX)

S4 Table Age-related changes in prevalence of cough in children at different age groups, presented as odds ratios (ORs) with 95% confidence intervals (CIs). (DOCX)

S5 Table Prevalence of cough triggered by food in children with and without reported reflux in infancy. (DOCX)

S6 Table Prevalence of cough in entire cohort, children with wheeze and without wheeze, stratified by sex. (DOCX)

S7 Table Prevalence of cough among children with current wheeze, by presence of doctor-diagnosed asthma, and by severity of wheeze (number of attacks). (DOCX)

S8 Table Unweighted and weighted prevalence of cough in the entire cohort of children, children with wheeze and children without wheeze. (DOCX)

S9 Table STROBE checklist for cohort studies. STROBE—Strengthening the reporting of observational studies in epidemiology.
(DOC)
