# Supplementary material for: Prevalence of cough throughout childhood: A cohort study
Source: PLoS One. 2017 May 24;12(5):e0177485. doi: 10.1371/journal.pone.0177485 (PMC5443519; doi:10.1371/journal.pone.0177485)
Supplement: S1 Text — Sensitivity statistical analysis. (DOCX) [file pone.0177485.s001.docx]

**S1 Text. Methods supporting information: Sensitivity statistical analysis**

Attrition bias, caused by decreasing response rates, is common in cohort studies. Since non-responders might comprise a different population than responders, attrition could distort the results, so that they are no longer representative for entire cohort. We explored whether attrition bias might have affected our results by using inverse probability weights, and comparing weighted prevalence of outcomes to unweighted prevalence. To do this, we modelled the probability of participation at each survey with logistic regression models, by assessing which baseline factors were associated with participation. The following baseline factors were tested: age; sex; ethnicity (white, South Asian); low birth weight (<2500 g); gestational age (<37 weeks); age of the mother at the sampling of participants; Townsend deprivation index ([1](#_ENREF_1)); living in an urban area (Leicester postcodes LE1-LE5) at first survey; and, participation in previous surveys. From these models we predicted individual response probabilities for different surveys, and inverted them to obtain individual weights ([2](#_ENREF_2)).

**References**

1. Townsend P, Philimore P, Beattie A. Health and deprivation: inequality and the North. Bristol: Croom Helm; 1988.

2. Austin PC. An Introduction to Propensity Score Methods for Reducing the Effects of Confounding in Observational Studies. Multivariate Behav Res. 2011 May;46(3):399-424. PubMed PMID: 21818162. Pubmed Central PMCID: Pmc3144483. Epub 2011/08/06. Eng.
