# Supplementary figures and images for: Prevalence of cough throughout childhood: A cohort study
Source: PLoS One. 2017 May 24;12(5):e0177485. doi: 10.1371/journal.pone.0177485 (PMC5443519; doi:10.1371/journal.pone.0177485)

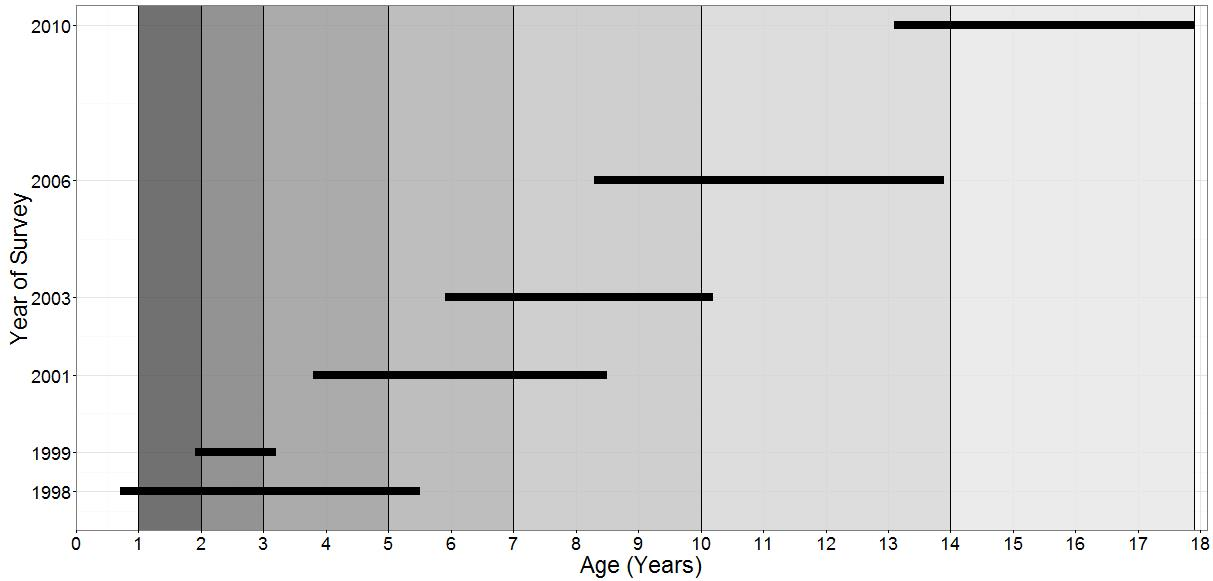

Supplement: S1 Fig — The bands on the x-axis show the seven age groups used for the analysis (1, 2, 3–4, 5–6, 7–9, 10–13 and 14-17-year-olds). The y-axis shows the years when surveys were conducted. In 1999, only those who responded in 1998 and were born in years 1996–7 (aged 1 year at the first survey) were addressed. (TIFF) [file pone.0177485.s002.tiff]

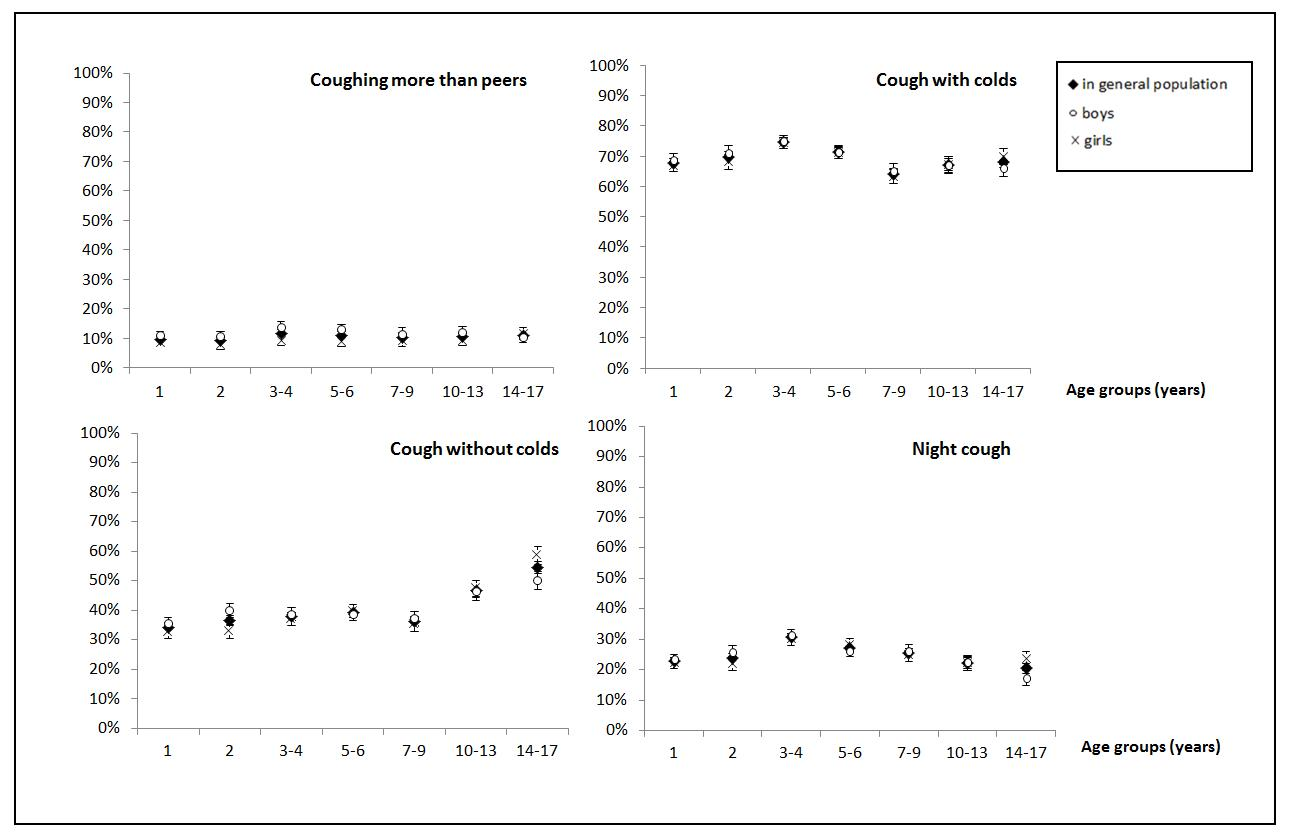

Supplement: S2 Fig — (TIF) [file pone.0177485.s003.tif]

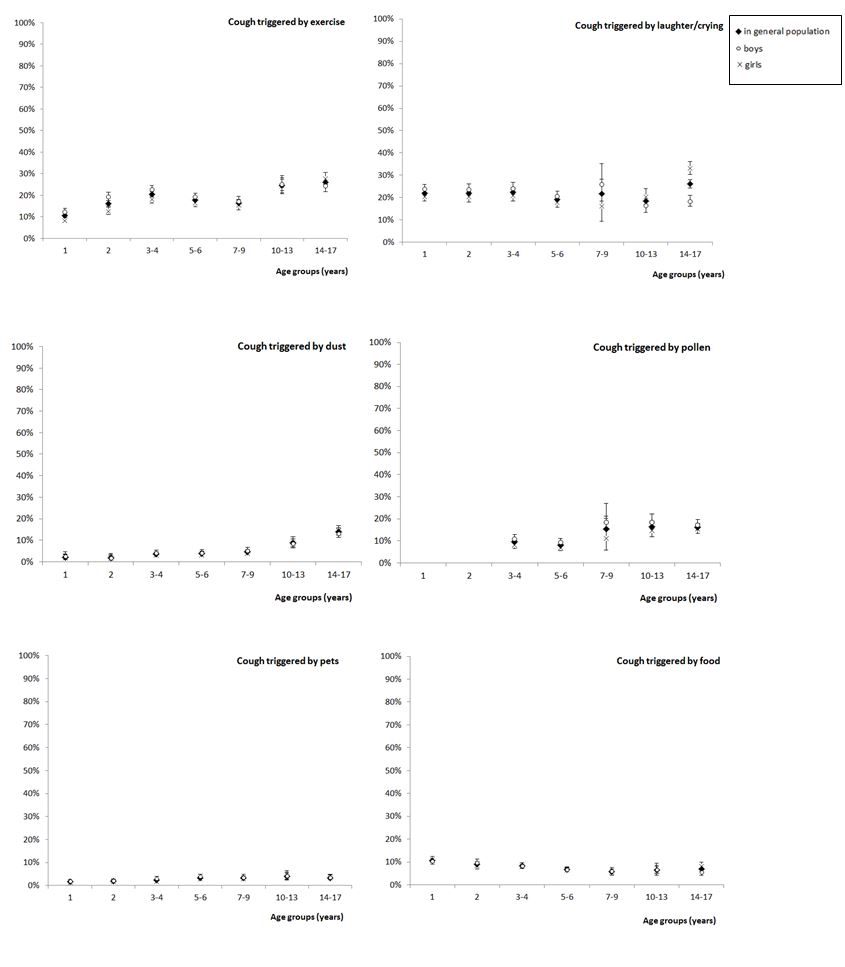

Supplement: S3 Fig — (JPG) [file pone.0177485.s004.JPG]
