# Supplementary material for: Prevalence of cough throughout childhood: A cohort study
Source: PLoS One. 2017 May 24;12(5):e0177485. doi: 10.1371/journal.pone.0177485 (PMC5443519; doi:10.1371/journal.pone.0177485)
Supplement: S1 Table — Number of children who were asked about certain aspects of cough in different surveys of Leicester Respiratory Cohorts and reported results with missing data, stratified by age group. (DOCX) [file pone.0177485.s005.docx]

**S1 Table. Total number of observations for questions on cough and information on missing data.** Number of children who were asked about certain aspects of cough in different surveys of Leicester Respiratory Cohorts and reported results with missing data, stratified by age group.

| **Age groups**  **Cough questions** | **1-year-olds**  **(N=4102)**  **n(%)** | **2-year-olds**  **(N=3163)**  **n(%)** | **3-4-year-olds**  **(N=4071)**  **n(%)** | **5-6-year-olds**  **(N=4031)**  **n(%)** | **7-9-year-olds**  **(N=3244)**  **n(%)** | **10-13-year-olds (N=2204)**  **n(%)** | **14-17-year-olds (N=2025)**  **n(%)** |
| --- | --- | --- | --- | --- | --- | --- | --- |
| Coughing more | 3277 | 2338 | 1899 | 2348 | 1438 | 2198 | 2025 |
| *Yes:* | 313 (9.55) | 213 (9.11) | 217 (11.43) | 257 (10.95) | 147 (10.22) | 227 (10.33) | 221 (10.92) |
| *No:* | 2893 (88.28) | 2055 (87.90) | 1647 (86.73) | 2058 (87.65) | 1274 (88.60) | 1950 (88.72) | 1785 (88.14) |
| *Missing:* | 71 (2.17) | 70 (2.99) | 35 (1.84) | 33 (1.41) | 17 (1.18) | 21 (0.96) | 19 (0.94) |
| Cough with colds | 4102 | 3163 | 4071 | 4031 | 3244 | 2204 | 2025 |
| *Yes:* | 2790 (68.02) | 2205 (69.71) | 3043 (74.75) | 2880 (71.45) | 2086 (64.30) | 1482 (67.24) | 1382 (68.18) |
| *No:* | 1252 (30.52) | 885 (27.98) | 979 (24.05) | 1094 (27.14) | 1118 (34.46) | 697 (31.62) | 618 (30.49) |
| *Missing:* | 60 (1.46) | 73 (2.31) | 49 (1.20) | 57 (1.41) | 40 (1.23) | 25 (1.13) | 25 (1.23) |
| Cough without colds | 4102 | 3163 | 4071 | 4031 | 3244 | 2204 | 2025 |
| *Yes:* | 1403 (34.20) | 1159 (36.64) | 1544 (37.93) | 1579 (39.17) | 1175 (36.22) | 1031 (46.78) | 1104 (54.52) |
| *No:* | 2654 (64.70) | 1931 (61.05) | 2461 (60.45) | 2381 (59.07) | 2008 (61.90) | 1142 (51.81) | 906 (44.74) |
| *Missing:* | 45 (1.10) | 73 (2.31) | 66 (1.62) | 71 (1.76) | 61 (1.88) | 31 (1.41) | 15 (0.74) |
| Night cough | 4102 | 3163 | 4071 | 4031 | 3244 | 2204 | 2025 |
| *Yes:* | 931 (22.70) | 750 (23.71) | 1245 (30.58) | 1088 (26.99) | 824 (25.40) | 486 (22.05) | 412 (20.35) |
| *No:* | 3117 (75.99) | 2339 (73.95) | 2757 (67.72) | 2887 (71.62) | 2368 (73.00) | 1657 (75.18) | 1556 (76.84) |
| *Missing:* | 54 (1.32) | 74 (2.34) | 69 (1.69) | 56 (1.39) | 52 (1.60) | 61 (2.77) | 57 (2.81) |
| *Cough triggers:* |  |  |  |  |  |  |  |
| Exercise/play | 4016 | 3067 | 3897 | 3896 | 1884 | 991 | 2025 |
| *Yes:* | 417 (10.38) | 494 (16.11) | 795 (20.40) | 695 (17.84) | 1563 (82.96) | 659 (66.50) | 1299 (64.15) |
| *No:* | 3241 (80.70) | 2356 (76.82) | 2963 (76.03) | 3017 (77.44) | 307 (16.30) | 244 (24.62) | 529 (26.12) |
| *Missing:* | 137 (3.41) | 112 (3.65) | 49 (1.26) | 56 (1.44) | 2 (0.11) | 28 (2.83) | 97 (4.79) |
| *Don’t know:* | 221 (5.50) | 105 (3.42) | 90 (2.31) | 128 (3.29) | 12 (0.64) | 60 (6.05) | 100 (4.94) |
| Laughter/cry | 3277 | 2338 | 1899 | 2348 | 189 | 985 | 2025 |
| *Yes:* | 728 (22.22) | 514 (21.98) | 427 (22.49) | 452 (19.25) | 41 (21.69) | 182 (18.48) | 529 (26.12) |
| *No:* | 2244 (68.48) | 1629 (69.67) | 1324 (69.72) | 1728 (73.59) | 138 (73.02) | 706 (71.68) | 1303 (64.35) |
| *Missing:* | 100 (3.05) | 99 (4.23) | 57 (3.00) | 68 (2.90) | 5 (2.65) | 49 (4.97) | 103 (5.09) |
| *Don’t know:* | 205 (6.26) | 96 (4.11) | 91 (4.79) | 100 (4.26) | 5 (2.65) | 48 (4.87) | 90 (4.44) |
| Dust | 825 | 825 | 2172 | 1683 | 1888 | 977 | 2025 |
| *Yes:* | 18 (2.18) | 14 (1.70) | 80 (3.68) | 63 (3.74) | 86 (4.56) | 82 (8.39) | 264 (13.03) |
| *No:* | 702 (85.09) | 684 (82.91) | 1819 (83.75) | 1414 (84.02) | 1570 (83.16) | 763 (78.10) | 1510 (74.57) |
| *Missing:* | 105 (12.73) | 127 (15.39) | 273 (12.57) | 206 (12.24) | 219 (11.60) | 34 (3.48) | 115 (5.68) |
| *Don’t know:* | - | - | - | - | 13 (0.69) | 98 (10.03) | 136 (6.72) |
| Pollen | - | - | 1846 | 2348 | 189 | 985 | 2025 |
| *Yes:* | - | - | 176 (9.53) | 191 (8.13) | 29 (15.34) | 163 (16.55) | 328 (16.20) |
| *No:* | - | - | 1381 (74.81) | 1815 (77.30) | 136 (71.96) | 712 (72.28) | 1494 (73.78) |
| *Missing:* | - | - | 67 (3.63) | 71 (3.02) | 4 (2.12) | 40 (4.06) | 96 (4.74) |
| *Don’t know:* | - | - | 222 (12.03) | 271 (11.54) | 20 (10.58) | 70 (7.11) | 107 (5.28) |
| Pets | 3927 | 3016 | 3988 | 3952 | 1993 | 977 | 2025 |
| *Yes:* | 61 (1.55) | 55 (1.82) | 95 (2.38) | 134 (3.39) | 64 (3.21) | 37 (3.79) | 65 (3.21) |
| *No:* | 3394 (86.43) | 2638 (87.47) | 3435 (86.13) | 3450 (87.30) | 1666 (83.59) | 828 (84.75) | 1754 (86.62) |
| *Missing:* | 112 (2.85) | 128 (4.24) | 297 (7.45) | 211 (5.34) | 241 (12.09) | 41 (4.20) | 122 (6.02) |
| *Don’t know:* | 360 (9.17) | 195 (6.47) | 161 (4.04) | 157 (3.97) | 22 (1.10) | 71 (7.27) | 84 (4.15) |
| Food/drinks | 3963 | 3036 | 3995 | 3960 | 1993 | 977 | 2025 |
| *Yes:* | 424 (10.70) | 276 (9.09) | 333 (8.34) | 263 (6.64) | 115 (5.77) | 60 (6.14) | 130 (6.42) |
| *No:* | 3136 (79.13) | 2487 (81.92) | 3296 (82.50) | 3333 (84.17) | 1699 (85.25) | 808 (82.70) | 1684 (83.16) |
| *Missing:* | 78 (1.97) | 97 (3.19) | 214 (5.36) | 166 (4.19) | 158 (7.93) | 40 (4.09) | 131 (6.47) |
| *Don’t know:* | 325 (8.20) | 176 (5.80) | 152 (3.80) | 198 (5.00) | 21 (1.05) | 69 (7.06) | 80 (3.95) |
|  |  |  |  |  |  |  |  |

N: number of children who returned the questionnaire at the respective age; n: number of children with positive, negative or missing information on cough at the respective age.
