# Supplementary material for: Prevalence of cough throughout childhood: A cohort study
Source: PLoS One. 2017 May 24;12(5):e0177485. doi: 10.1371/journal.pone.0177485 (PMC5443519; doi:10.1371/journal.pone.0177485)
Supplement: S2 Table — (DOCX) [file pone.0177485.s006.docx]

**S2 Table. Prevalence of wheeze and doctor-diagnosed asthma in different age groups.**

| Age (years) | **Current wheeze**  **(last 12 months)** | | | **Doctor-diagnosed asthma**  **(ever in life)** | | | |
| --- | --- | --- | --- | --- | --- | --- | --- |
|  | n/N | % | 95% CI | | n/N | % | 95% CI |
| 1 | 1409/4102 | 34 | 33 – 36 | | 103/843 | 12 | 10 – 15 |
| 2 | 726/3163 | 23 | 22 – 25 | | 435/3020 | 14 | 13 – 16 |
| 3-4 | 761/4071 | 19 | 18 – 20 | | 798/3985 | 20 | 19 – 21 |
| 5-6 | 606/4031 | 15 | 14 – 16 | | 853/3998 | 21 | 20 – 23 |
| 7-9 | 434/3244 | 13 | 12 – 15 | | 725/3228 | 22 | 21 – 24 |
| 10-13 | 331/2204 | 15 | 14 – 17 | | 459/2204 | 21 | 19 – 23 |
| 14-17 | 309/2025 | 15 | 14 – 17 | | 471/2025 | 23 | 21 – 25 |

n: number of children with wheeze or doctor-diagnosed asthma ever at the respective age;

N: number of children who returned the questionnaire at the respective age;

CI: confidence interval.
