# Supplementary material for: Prevalence of cough throughout childhood: A cohort study
Source: PLoS One. 2017 May 24;12(5):e0177485. doi: 10.1371/journal.pone.0177485 (PMC5443519; doi:10.1371/journal.pone.0177485)
Supplement: S3 Table — (DOCX) [file pone.0177485.s007.docx]

**S3 Table. Prevalence of doctor-diagnosed asthma (ever) in different age groups, stratified by current wheeze.**

| Prevalence of doctor-diagnosed asthma | **Current wheeze** | | **No current wheeze** | |
| --- | --- | --- | --- | --- |
|  |  |  |  |  |
| Age (years) | n/N | % [95%CI] | n/N | % [95%CI] |
| 1 | 85/254 | 33 [28-40] | 18/589 | 3 [2-5] |
| 2 | 294/679 | 43 [40-47] | 141/2341 | 6 [5-7] |
| 3-4 | 440/742 | 59 [56-63] | 358/3243 | 11 [10-12] |
| 5-6 | 406/602 | 67 [64-71] | 447/3396 | 13 [12-14] |
| 7-9 | 298/431 | 69 [65-73] | 427/2797 | 15 [14-17] |
| 10-13 | 201/331 | 61 [55-66] | 258/1873 | 14 [12-15] |
| 14-17 | 198/308 | 64 [59-69] | 273/1715 | 16 [14-18] |

n: number of children with doctor-diagnosed asthma ever at the respective age;

N: number of children who returned the questionnaire at the respective age;

CI: confidence interval.
