# Supplementary material for: Prevalence of cough throughout childhood: A cohort study
Source: PLoS One. 2017 May 24;12(5):e0177485. doi: 10.1371/journal.pone.0177485 (PMC5443519; doi:10.1371/journal.pone.0177485)
Supplement: S4 Table — (DOCX) [file pone.0177485.s008.docx]

| **S4 Table. Age-related changes in prevalence of cough in children at different age groups, presented as odds ratios (ORs) with 95% confidence intervals (CIs).** | | | | | | | |
| --- | --- | --- | --- | --- | --- | --- | --- |
| **Age group** | **1 years** | **2 years** | **3-4 years** | **5-6 years** | **7-9 years** | **10-13 years** | **14-17 years** |
| Cough | Age-related change^£^ | Age-related change^£^ | Age-related change^£^ | Age-related change^£^ | Age-related change^£^ | Age-related change^£^ | Age-related change^£^ |
| *Entire cohort* | baseline OR | OR[95%CI], p-value | OR[95%CI], p-value | OR[95%CI], p-value | OR[95%CI], p-value | OR[95%CI], p-value | OR[95%CI], p-value |
| Coughing more^#^ | 1 | 1.0[0.8-1.1], 0.669 | 1.3[1.1-1.5], 0.001 | 1.2[1.0-1.3], 0.043 | 1.1[0.9-1.3], 0.473 | 1.1[0.9-1.3], 0.308 | 1.2[1.0-1.4], 0.049 |
| Cough with colds | 1 | 1.1[1.0-1.2], 0.088 | 1.4[1.3-1.5], <0.001 | 1.2[1.1-1.3], <0.001 | 0.8[0.8-0.9], <0.001 | 1.0[0.9-1.1], 0.378 | 1.0[0.9-1.1], 0.757 |
| Cough without colds | 1 | 1.1[1.0-1.2], 0.002 | 1.4[1.3-1.5], <0.001 | 1.4[1.3-1.5], <0.001 | 1.3[1.2-1.4], <0.001 | 2.1[1.9-2.3], <0.001 | 2.7[2.4-3.0], <0.001 |
| Night cough^¶^ | 1 | 1.1[1.0-1.2], 0.187 | 1.5[1.3-1.6], <0.001 | 1.2[1.1-1.3], <0.001 | 1.2[1.0-1.3], 0.003 | 1.0[0.9-1.1], 0.582 | 0.9[0.8-1.0], 0.029 |
| *Cough triggers:*^¶^ |  |  |  |  |  |  |  |
| Exercise/play ^#^ | 1 | 1.7[1.5-1.9], <0.001 | 2.3[2.0-2.6], <0.001 | 1.9[1.7-2.1], <0.001 | 1.8[1.6-2.1], <0.001 | 2.9[2.4-3.4], <0.001 | 3.2[2.8-3.7], <0.001 |
| Laughter/crying^#^ | 1 | 1.0[0.9-1.1], 0.994 | 1.1[0.9-1.2], 0.373 | 0.8[0.7-1.0], 0.008 | 0.9[0.6-1.2], 0.367 | 0.9[0.6-1.0], 0.019 | 1.3[1.1-1.4], <0.001 |
| Dust^#^ | 1 | 0.8[0.4-1.5], 0.480 | 1.6[1.0-2.6], 0.052 | 1.7[1.0-2.8], 0.033 | 2.1[1.3-3.5], 0.002 | 4.3[2.6-7.0], <0.001 | 6.8[4.3-10.7], <0.001 |
| Pollen^#^ | - | - | 1 | 0.8[0.7-1.0], 0.048 | 1.6[1.1-2.4], 0.022 | 1.8[1.5-2.2], <0.001 | 1.9[1.6-2.3], <0.001 |
| Pets^#^ | 1 | 1.2[0.9-1.7], 0.283 | 1.6[1.2-2.1], 0.003 | 2.1[1.6-3.1], <0.001 | 2.2[1.6-3.1], <0.001 | 2.7[1.9-4.0], <0.001 | 2.3[1.7-3.2], <0.001 |
| Food/drinks^#^ | 1 | 0.9[0.7-1.0], 0.038 | 0.7[0.6-0.9], <0.001 | 0.6[0.5-0.7], <0.001 | 0.5[0.4-0.6], <0.001 | 0.6[0.5-0.8], <0.001 | 0.6[0.5-0.7], <0.001 |
| *Wheezers* | baseline OR | OR[95%CI], p-value | OR[95%CI], p-value | OR[95%CI], p-value | OR[95%CI], p-value | OR[95%CI], p-value | OR[95%CI], p-value |
| Coughing more^#^ | 1 | 1.2[1.0-1.5], 0.045 | 1.9[1.5-2.4], <0.001 | 2.3[1.8-2.8], <0.001 | 1.8[1.3-2.3], <0.001 | 2.3[1.8-2.9], <0.001 | 1.8[1.4-2.4], <0.001 |
| Cough with colds | 1 | 1.4[1.1-1.8], 0.012 | 1.9[1.5-2.5], <0.001 | 1.8[1.3-2.3], <0.001 | 1.2[0.9-1.6], 0.181 | 1.3[0.9-1.8], 0.133 | 1.1[0.8-1.6], 0.573 |
| Cough without colds | 1 | 1.2[1.0-1.5], 0.010 | 1.6[1.4-1.9], <0.001 | 1.7[1.4-2.0], <0.001 | 2.1[1.7-2.6], <0.001 | 3.2[2.5-4.1], <0.001 | 3.0[2.3-4.0], <0.001 |
| Night cough^¶^ | 1 | 1.4[1.2-1.6], <0.001 | 2.1[1.8-2.5], <0.001 | 1.9[1.6-2.3], <0.001 | 2.2[1.8-2.7], <0.001 | 1.9[1.5-2.4], <0.001 | 1.2[1.0-1.6], 0.100 |
| *Cough triggers:*^¶^ |  |  |  |  |  |  |  |
| Exercise/play ^#^ | 1 | 2.2[1.9-2.7], <0.001 | 3.6[3.0-4.3], <0.001 | 3.8[3.2-4.6], <0.001 | 4.4[3.5-5.7], <0.001 | 4.7[3.5-6.2], <0.001 | 5.6[4.4-7.3], <0.001 |
| Laughter/crying^#^ | 1 | 1.2[1.0-1.5], 0.026 | 1.3[1.0-1.6], 0.020 | 1.6[1.3-2.0], <0.001 | 1.5[0.9-2.5], 0.165 | 1.2[0.9-1.6], 0.349 | 1.3[1.0-1.6], 0.065 |
| Dust^#^ | 1 | 1.5[0.6-3.4], 0.376 | 2.7[1.4-5.3], 0.004 | 3.3[1.7-6.5], 0.001 | 3.9[2.0-7.7], <0.001 | 7.2[3.6-14.4], <0.001 | 10.6[5.5-20.4], <0.001 |
| Pollen^#^ | - | - | 1 | 1.0[0.7-1.3], 0.886 | 1.6[0.9-2.9], 0.090 | 1.8[1.2-2.5], 0.002 | 1.5[1.1-2.1], 0.015 |
| Pets^#^ | 1 | 1.6[1.1-2.3], 0.022 | 2.5[1.7-3.5], <0.001 | 4.1[3.0-5.7], <0.001 | 4.6[3.1-6.8], <0.001 | 4.2[2.6-6.8], <0.001 | 3.3[2.1-5.1], <0.001 |
| Food/drinks^#^ | 1 | 0.8[0.6-1.1], 0.184 | 0.9[0.7-1.1], 0.488 | 1.0[0.7-1.3], 0.883 | 1.2[0.8-1.7], 0.336 | 1.3[0.8-2.0], 0.251 | 0.7[0.5-1.1], 0.129 |
| *Non-wheezers* | baseline OR | OR[95%CI], p-value | OR[95%CI], p-value | OR[95%CI], p-value | OR[95%CI], p-value | OR[95%CI], p-value | OR[95%CI], p-value |
| Coughing more^#^ | 1 | 1.3[0.9-1.7], 0.123 | 2.0[1.5-2.7], <0.001 | 1.8[1.4-2.3], <0.001 | 1.8[1.3-2.5], <0.001 | 1.6[1.2-2.2], 0.001 | 2.0[1.5-2.7], <0.001 |
| Cough with colds | 1 | 1.2[1.1-1.3], <0.001 | 1.6[1.5-1.8], <0.001 | 1.4[1.3-1.5], <0.001 | 1.0[0.9-1.1], 0.954 | 1.1[1.0-1.3], 0.026 | 1.2[1.1-1.3], 0.004 |
| Cough without colds | 1 | 1.3[1.2-1.5], <0.001 | 1.6[1.4-1.8], <0.001 | 1.7[1.5-1.9], <0.001 | 1.5[1.4-1.7], <0.001 | 2.5[2.2-2.8], <0.001 | 3.4[3.0-3.9], <0.001 |
| Night cough^¶^ | 1 | 1.1[1.0-1.3], 0.056 | 1.7[1.5-1.9], <0.001 | 1.5[1.3-1.6], <0.001 | 1.3[1.2-1.5], <0.001 | 1.0[0.9-1.2], 0.703 | 1.0[0.9-1.2], 0.677 |
| *Cough triggers:*^¶^ |  |  |  |  |  |  |  |
| Exercise/play ^#^ | 1 | 2.2[1.8-2.7], <0.001 | 3.3[2.7-4.0], <0.001 | 2.7[2.2-3.2], <0.001 | 2.5[2.0-3.1], <0.001 | 4.2[3.3-5.4], <0.001 | 5.1[4.1-6.2], <0.001 |
| Laughter/crying^#^ | 1 | 1.1[0.9-1.3], 0.110 | 1.3[1.1-1.5], 0.006 | 0.9[0.8-1.1], 0.476 | 0.7[0.4-1.2], 0.251 | 0.9[0.7-1.1], 0.394 | 1.8[1.5-2.1], <0.001 |
| Dust^#^ | 1 | 0.4[0.1-1.3], 0.134 | 1.5[0.7-3.1], 0.316 | 1.4[0.7-3.0], 0.354 | 2.3[1.1-4.7], 0.026 | 4.2[2.0-8.8], <0.001 | 8.0[4.0-16.1], <0.001 |
| Pollen^#^ | - | - | 1 | 0.8[0.6-1.0], 0.152 | 1.2[0.6-2.3], 0.678 | 1.9[1.4-2.5], <0.001 | 2.4[1.9-3.1], <0.001 |
| Pets^#^ | 1 | 1.2[0.6-2.4], 0.678 | 1.7[0.9-3.2], 0.105 | 2.2[1.2-4.0], 0.009 | 3.0[1.6-5.6], <0.001 | 3.3[1.5-6.9], 0.002 | 3.4[1.8-6.5], <0.001 |
| Food/drinks^#^ | 1 | 0.9[0.7-1.1], 0.165 | 0.7[0.6-0.9], 0.001 | 0.6[0.5-0.7], <0.001 | 0.4[0.3-0.6], <0.001 | 0.5[0.3-0.6], <0.001 | 0.6[0.5-0.7], <0.001 |
| OR: odds ratio; CI: confidence interval;  ^#^: only asked in part of the cohort;  ^¶^: symptoms occurring in the past 12 months;  ^£^: age-related change – test for the significance of change in prevalence with age for each of the symptoms, calculated with generalised estimating equation (GEE) and presented as odds ratio (OR) of change in prevalence per age group with respective p-values, accounting for repeated nature of measurements and clustered data (Baseline group: 1-year-old children). | | | | | | | |
|  | | | | |  |  |  |
