# Supplementary material for: Prevalence of cough throughout childhood: A cohort study
Source: PLoS One. 2017 May 24;12(5):e0177485. doi: 10.1371/journal.pone.0177485 (PMC5443519; doi:10.1371/journal.pone.0177485)
Supplement: S5 Table — (DOCX) [file pone.0177485.s009.docx]

**S5 Table. Prevalence of cough triggered by food in children with and without reported reflux in infancy.**

| Prevalence of cough triggered by food | **Gastroesophageal reflux** | | **No gastroesophageal reflux** | | | **P-value^*^** |
| --- | --- | --- | --- | --- | --- | --- |
|  | **in infancy** |  | **in infancy** | |  |  |
| Age (years) | n/N | % [95%CI] | n/N | % [95%CI] |  |  |
| 1 | 108/634 | 17 [14-20] | 60/991 | 6 [5-8] |  | <0.001 |
| 2 | 26/129 | 20 [14-28] | 15/217 | 7 [4-11] |  | <0.001 |
| 3-4 | 46/244 | 19 [14-24] | 26/490 | 5 [4-8] |  | <0.001 |

n: number of children with cough triggered by food at the respective age; N: number of children who returned the questionnaire

at the respective age; CI: confidence interval;

^*^: p-value of Chi-square test for association between reflux and cough triggered by food.
