# Supplementary material for: Prevalence of cough throughout childhood: A cohort study
Source: PLoS One. 2017 May 24;12(5):e0177485. doi: 10.1371/journal.pone.0177485 (PMC5443519; doi:10.1371/journal.pone.0177485)
Supplement: S6 Table — (DOCX) [file pone.0177485.s010.docx]

**S6 Table. Prevalence of cough in entire cohort, children with wheeze and without wheeze, stratified by sex.**

| **Age group** | **1-year-olds**  **(N=4102)** | | | | **2-year-olds**  **(N=3163)** | | | | **3-4-year-olds**  **(N=4071)** | | **5-6-year-olds**  **(N=4031)** | | **7-9-year-olds**  **(N=3244)** | | **10-13-year-olds (N=2204)** | | **14-17-year-olds (N=2025)** | |
| --- | --- | --- | --- | --- | --- | --- | --- | --- | --- | --- | --- | --- | --- | --- | --- | --- | --- | --- |
| *Entire cohort* | Boys  (n=2136) %[CI] | | Girls  (n=1966)  %[CI] | | Boys  (n=1641) %[CI] | | | Girls  (n=1522)  %[CI] | Boys  (n=2102) %[CI] | Girls  (n=1969)  %[CI] | Boys  (n=2126) %[CI] | Girls  (n=1905)  %[CI] | Boys  (n=1691) %[CI] | Girls  (n=1553)  %[CI] | Boys  (n=1083) %[CI] | Girls  (n=1121)  %[CI] | Boys  (n=970) %[CI] | Girls  (n=1055)  %[CI] |
| Coughing more^#^ | ***11[9-12]** | | **8[7-10]** | | ***10[9-12]** | | | **8[6-9]** | ***14[11-16]** | **9[8-11]** | ***13[11-15]** | **9[7-11]** | 11[9-14] | 9[7-12] | ***12[10-14]** | **9[7-11]** | 10[8-12] | 12[10-14] |
| Cough with colds | 69[67-71] | | 67[65-69] | | 71[69-73] | | | 68[66-70] | 75[73-77] | 74[72-76] | 72[70-74] | 71[69-73] | 65[63-68] | 63[61-66] | 67[64-70] | 67[64-70] | 66[63-69] | 70[67-73] |
| Cough without colds | ***36[34-38]** | | **33[31-35]** | | ***40[38-42]** | | | **33[31-35]** | 39[37-41] | 37[35-39] | 39[37-41]] | 40[38-42] | 37[35-39] | 35[33-38] | 46[43-49] | 47[44-50] | ***50[47-53]** | **59[56-61]** |
| Night cough^¶^ | 23[22-25] | | 22[20-24] | | ***26[24-28]** | | | **22[20-24]** | 31[29-33] | 30[28-32] | 26[24-28] | 28[26-30] | 26[24-28] | 25[23-27] | 22[20-25] | 22[20-24] | ***17[15-20]** | **23[21-26]** |
| *Cough triggers*^¶^*:* |  | |  | |  | | |  |  |  |  |  |  |  |  |  |  |  |
| Exercise/play^#^ | ***12[11-14]** | | **8[7-10]** | | ***19[17-21]** | | | **13[11-14]** | ***23[21-25]** | **18[16-20]** | ***19[18-21]** | **16[15-18]** | 17[15-20] | 15[13-18] | 25[21-29] | 24[21-28] | 24[22-27] | 28[25-30] |
| Laughter/crying^#^ | ***24[22-26]** | | **20[18-22]** | | 24[21-26] | | | 20[18-22] | 24[21-27] | 21[18-24] | 21[18-23] | 18[16-20] | 26[18-35] | 16[9-26] | 16[13-20] | 20[17-24] | ***18[16-21]** | **33[30-36]** |
| Dust^#^ | 3[1-5] | | 2[1-4] | | 2[1-3] | | | 1[1-3] | 4[3-5] | 3[2-4] | 4[3-6] | 3[2-5] | 5[4-7] | 4[3-5] | 9[6-12] | 9[7-12] | 13[11-16] | 14[12-17] |
| Pollen^#^ | - | | - | | - | | | - | ***11[9-13]** | **8[7-10]** | ***9[8-11]** | **7[5-8]** | 19[12-27] | 11[6-20] | 19[15-22] | 15[12-18] | 17[15-20] | 15[13-18] |
| Pets^#^ | ***2[1-3]** | | **1[1-2]** | | 2[1-3] | | | 2[1-2] | ***3[2-4]** | **2[1-2]** | 4[3-5] | 3[2-4] | 4[3-5] | 3[2-4] | 4[3-7] | 4[2-6] | 3[2-5] | 3[2-5] |
| Food/drinks^#^ | 10[9-12] | | 11[10-13] | | 10[8-11] | | | 8[7-10] | 8[7-10] | 8[7-10] | 7[6-8] | 7[6-8] | 6[5-8] | 5[4-7] | 7[5-10] | 6[4-8] | ***6[4-7]** | **8[7-10]** |
|  |  | |  | |  |  | | |  |  |  |  |  |  |  |  |  |  |
|  | **1-year-olds**  **(N=1409)** | | | | **2-year-olds**  **(N=726)** | | | | **3-4-year-olds**  **(N=761)** | | **5-6-year-olds**  **(N=606)** | | **7-9-year-olds**  **(N=434)** | | **10-13-year-olds (N=331)** | | **14-17-year-olds (N=309)** | |
| *Wheezers* | Boys  (n=788) %[CI] | | Girls  (n=621)  %[CI] | | Boys  (n=414) %[CI] | | Girls  (n=312)  %[CI] | | Boys  (n=431) %[CI] | Girls  (n=330)  %[CI] | Boys  (n=355) %[CI] | Girls  (n=251)  %[CI] | Boys  (n=270) %[CI] | Girls  (n=164)  %[CI] | Boys  (n=202) %[CI] | Girls  (n=129)  %[CI] | Boys  (n=148) %[CI] | Girls  (n=161)  %[CI] |
| Coughing more^#^ | 23[20-26] | | 20[17-24] | | 29[25-35] | | 25[19-31] | | 39[32-46] | 31[24-39] | ***46[40-53]** | **35[27-43]** | 36[28-44] | 32[22-43] | 41[34-48] | 35[27-44] | 31[24-39] | 36[29-44] |
| Cough with colds | 85[82-87] | | 83[80-86] | | 89[85-92] | | 87[83-90] | | 91[88-94] | 90[87-93] | 90[86-93] | 92[87-94] | 87[82-90] | 88[82-93] | 87[81-92] | 87[80-92] | 87[81-92] | 83[77-88] |
| Cough without colds | 52[48-55] | | 50[46-54] | | ***60[55-64]** | | **52[46-57]** | | 62[57-66] | 62[57-68] | 68[63-72] | 63[57-69] | 67[61-72] | 71[63-77] | 71[65-77] | 81[73-87] | 70[62-77] | 78[71-84] |
| Night cough^¶^ | 37[34-41] | | 34[31-38] | | 45[41-50] | | 42[37-48] | | 55[50-59] | 56[50-61] | 54[48-59] | 52[46-58] | 54[48-60] | 62[54-69] | 49[42-55] | 57[49-66] | 35[27-43] | 44[37-52] |
| *Cough triggers*^¶^*:* |  | |  | |  | |  | |  |  |  |  |  |  |  |  |  |  |
| Exercise/play^#^ | ***25[22-28]** | | **18[15-21]** | | 42[37-46] | | 34[29-40] | | 51[46-56] | 49[44-55] | 55[50-60] | 51[45-57] | 56[48-64] | 57[47-66] | 57[47-66] | 58[47-68] | 66[58-73] | 57[49-65] |
| Laughter/crying^#^ | 37[34-41] | | 34[30-38] | | 43[38-49] | | 38[32-45] | | 43[36-50] | 44[36-52] | 49[42-55] | 46[38-54] | 48[31-66] | 40[20-64] | 37[28-47] | 39[29-50] | **35[28-44]** | **48[40-56]** |
| Dust^#^ | 4[1-8] | | 4[2-10] | | 6[3-12] | | 5[2-11] | | 11[8-16] | 9[6-14] | 14[9-21] | 14[8-21] | 17[11-24] | 13[7-22] | 22[15-32] | 23[15-33] | 31[24-39] | 33[25-41] |
| Pollen^#^ | - | | - | | - | | - | | ***32[26-39]** | **20[14-27]** | 28[23-35] | 24[17-31] | 39[24-58] | 35[16-60] | 41[32-51] | 41[31-52] | 35[28-44] | 33[26-41] |
| Pets^#^ | 4[3-6] | | 3[2-4] | | 7[4-9] | | 5[3-8] | | 11[8-14] | 7[4-10] | 18[14-22] | 13[9-18] | 15[10-21] | 14[8-22] | 13[8-22] | 14[8-24] | 15[10-22] | 9[8-15] |
| Food/drinks^#^ | 14[9-21] | | 11[9-14] | | 10[7-13] | | 10[7-14] | | 10[8-14] | 12[9-16] | 13[9-17] | 11[8-16] | 16[11-22] | 15[9-23] | 15[9-24] | 14[8-23] | 7[4-13] | 14[10-21] |
|  |  |  | | |  | |  | |  |  |  |  |  |  |  |  |  |  |
|  | **1-year-olds**  **(N=2693)** | | | | **2-year-olds**  **(N=2437)** | | | | **3-4-year-olds**  **(N=3310)** | | **5-6-year-olds**  **(N=3425)** | | **7-9-year-olds**  **(N=2810)** | | **10-13-year-olds (N=1873)** | | **14-17-year-olds (N=1716)** | |
| *Non-wheezers* | Boys  (n=1348) %[CI] | | | Girls  (n=1345)  %[CI] | Boys  (n=1227) %[CI] | | | Girls  (n=1210)  %[CI] | Boys  (n=1671) %[CI] | Girls  (n=1639)  %[CI] | Boys  (n=1771) %[CI] | Girls  (n=1654)  %[CI] | Boys  (n=1421) %[CI] | Girls  (n=1389)  %[CI] | Boys  (n=881) %[CI] | Girls  (n=992)  %[CI] | Boys  (n=822) %[CI] | Girls  (n=894)  %[CI] |
| Coughing more^#^ | 3[2-5] | | | 3[2-4] | 4[3-5] | | | 4[3-5] | 7[6-9] | 5[4-7] | 6[5-8] | 5[4-7] | 5[4-7] | 6[5-8] | 5[4-7] | 6[4-7] | 6[5-8] | 7[6-9] |
| Cough with colds | 60[57-62] | | | 60[57-62] | 65[63-68] | | | 63[60-66] | 71[68-73] | 71[69-73] | 68[66-70] | 68[66-70] | 61[59-64] | 60[58-63] | 63[59-66] | 65[62-68] | ***63[59-66]** | **68[64-71]** |
| Cough without colds | 26[24-29] | | | 25[22-27] | ***33[31-36]** | | | **28[26-31]** | 33[31-35] | 32[30-34] | ***33[31-35]** | **36[34-39]** | 31[29-34] | 31[29-34] | 41[37-44] | 43[40-46] | ***46[43-50]** | **55[52-58]** |
| Night cough^¶^ | 15[13-17] | | | 16[15-19] | 19[17-21] | | | 16[14-18] | 25[23-27] | 25[23-27] | ***20[19-22]** | **24[22-27]** | 21[19-23] | 20[18-23] | 16[14-19] | 17[15-20] | ***14[12-17]** | **19[17-22]** |
| *Cough triggers*^¶^*:* |  | | |  |  | | |  |  |  |  |  |  |  |  |  |  |  |
| Exercise/play^#^ | 5[4-7] | | | 4[3-5] | ***12[10-14]** | | | **7[6-9]** | ***15[14-17]** | **12[10-13]** | 12[11-14] | 11[10-13] | 10[8-12] | 10[8-13] | 16[13-20] | 18[14-22] | ***17[14-20]** | **22[20-25]** |
| Laughter/crying^#^ | 16[14-18] | | | 14[12-16] | 17[15-19] | | | 16[14-19] | 19[17-22] | 17[14-19] | 15[13-17] | 14[12-16] | 16[9-26] | 8[3-19] | ***11[8-14]** | **17[13-21]** | ***15[13-18]** | **31[28-34]** |
| Dust^#^ | 2[1-5] | | | 0.7[0.2-2.9] | 0.6[0.2-2.5] | | | 0.3[0.04-2.4] | 2[1-3] | 2[1-3] | 2[2-4] | 1[1-3] | 3[2-5] | 3[2-4] | 5[3-8] | 6[4-9] | 10[8-12] | 11[9-13] |
| Pollen^#^ | - | | | - | - | | | - | 6[4-8] | 6[5-8] | 5[4-7] | 4[3-6] | 9[4-19] | 3[1-13] | 12[9-16] | 9[7-13] | 14[12-16] | 12[10-15] |
| Pets^#^ | 0.6[0.3-1.2] | | | 0.4[0.2-1.0] | 0.5[0.2-1.1] | | | 0.7[0.3-1.4] | 1.1[0.7-1.7] | 0.7[0.4-1.2] | 1[1-2] | 1[1-2] | 1.5[1-3] | 1[1-3] | 2[1-4] | 2[1-4] | 1[1-3] | 2[2-3] |
| Food/drinks^#^ | 9[8-11] | | | 11[9-13] | 10[8-12] | | | 8[6-10] | 8[7-9] | 8[6-9] | 6[5-7] | 6[5-7] | 4[3-6] | 4[3-6] | 5[3-7] | 4[3-7] | 5[4-7] | 7[5-9] |
|  |  | | |  |  | | |  |  |  |  |  |  |  |  |  |  |  |

CI: confidence interval; ^#^: only asked in part of the cohort; ^¶^: symptoms occurring in the past 12 months; *: significant difference in cough prevalence between boys and girls (in bold), calculated with likelihood ratio tests, p<0.05.
