# Supplementary material for: Prevalence of cough throughout childhood: A cohort study
Source: PLoS One. 2017 May 24;12(5):e0177485. doi: 10.1371/journal.pone.0177485 (PMC5443519; doi:10.1371/journal.pone.0177485)
Supplement: S7 Table — (DOCX) [file pone.0177485.s011.docx]

**S7 Table. Prevalence of cough among children with current wheeze, by presence of doctor-diagnosed asthma, and by severity of wheeze (number of attacks).**

| **Age group** | **1-year-olds** | | **2-year-olds** | | **3-4-year-olds** | | **5-6-year-olds** | | **7-9-year-olds** | | **10-13-year-olds** | | **14-17-year-olds** | |
| --- | --- | --- | --- | --- | --- | --- | --- | --- | --- | --- | --- | --- | --- | --- |
| *Coughing more* | %[CI] |  | %[CI] |  | %[CI] |  | %[CI] |  | %[CI] |  | %[CI] |  | %[CI] |  |
| Asthma diagnosis | -* |  | 44[38-51] |  | 45[38-52] |  | 49[43-55] |  | 42[35-50] |  | 46[39-53] |  | 39[32-46] |  |
| No asthma diagnosis | -* |  | 14[10-19] |  | 20[14-27] |  | 24[16-33] |  | 19[11-29] |  | 27[20-35] |  | 25[17-34] |  |
|  |  |  |  |  |  |  |  |  |  |  |  |  |  |  |
| Frequent wheeze^#^ | 39[34-44] |  | 47[39-55] |  | 51[41-61] |  | 63[54-71] |  | 38[28-48] |  | 55[46-64] |  | 44[34-54] |  |
| Infrequent wheeze^¶^ | 13[11-16] |  | 19[15-23] |  | 28[23-34] |  | 31[26-37] |  | 32[24-41] |  | 30[24-36] |  | 29[23-36] |  |
|  |  |  |  |  |  |  |  |  |  |  |  |  |  |  |
| *Cough with colds* |  |  |  |  |  |  |  |  |  |  |  |  |  |  |
| Asthma diagnosis | 91[82-95] |  | 91[88-94] |  | 92[89-94] |  | 91[88-94] |  | 89[85-92] |  | 89[84-93] |  | 85[80-90] |  |
| No asthma diagnosis | 82[75-87] |  | 85[81-88] |  | 89[85-92] |  | 89[83-93] |  | 83[76-89] |  | 85[77-90] |  | 85[76-90] |  |
|  |  |  |  |  |  |  |  |  |  |  |  |  |  |  |
| Frequent wheeze^#^ | 89[86-92] |  | 94[90-96] |  | 91[88-94] |  | 91[87-94] |  | 90[85-94] |  | 81[73-88] |  | 82[73-88] |  |
| Infrequent wheeze^¶^ | 81[78-84] |  | 85[81-88] |  | 91[88-93] |  | 90[87-93] |  | 85[80-89] |  | 90[86-94] |  | 87[81-91] |  |
|  |  |  |  |  |  |  |  |  |  |  |  |  |  |  |
| *Cough without colds* |  |  |  |  |  |  |  |  |  |  |  |  |  |  |
| Asthma diagnosis | 45[34-56] |  | 69[63-74] |  | 67[62-71] |  | 73[68-77] |  | 72[67-77] |  | 81[74-86] |  | 77[70-82] |  |
| No asthma diagnosis | 26[20-33] |  | 47[42-52] |  | 57[51-62] |  | 51[44-58] |  | 59[51-68] |  | 66[57-74] |  | 70[61-78] |  |
|  |  |  |  |  |  |  |  |  |  |  |  |  |  |  |
| Frequent wheeze^#^ | 66[62-70] |  | 69[63-74] |  | 68[62-73] |  | 74[68-80] |  | 76[70-82] |  | 78[69-85] |  | 74[65-82] |  |
| Infrequent wheeze^¶^ | 42[39-46] |  | 50[46-54] |  | 58[54-63] |  | 60[55-65] |  | 61[55-68] |  | 74[67-79] |  | 74[68-80] |  |
|  |  |  |  |  |  |  |  |  |  |  |  |  |  |  |
| *Night cough* |  |  |  |  |  |  |  |  |  |  |  |  |  |  |
| Asthma diagnosis | 52[41-62] |  | 57[51-62] |  | 59[55-64] |  | 61[56-65] |  | 61[55-66] |  | 54[47-61] |  | 44[38-51] |  |
| No asthma diagnosis | 31[24-38] |  | 35[30-40] |  | 50[44-55] |  | 37[31-44] |  | 48[40-57] |  | 49[41-58] |  | 31[23-40] |  |
|  |  |  |  |  |  |  |  |  |  |  |  |  |  |  |
| Frequent wheeze^#^ | 48[44-52] |  | 58[52-64] |  | 66[61-71] |  | 67[61-73] |  | 65[58-71] |  | 59[49-68] |  | 51[41-61] |  |
| Infrequent wheeze^¶^ | 29[26-32] |  | 37[32-41] |  | 48[43-52] |  | 43[38-48] |  | 50[43-56] |  | 48[42-55] |  | 34[28-41] |  |
|  |  |  |  |  |  |  |  |  |  |  |  |  |  |  |
| *Cough triggers* |  |  |  |  |  |  |  |  |  |  |  |  |  |  |
| *Exercise/play* |  |  |  |  |  |  |  |  |  |  |  |  |  |  |
| Asthma diagnosis | 29[20-41] |  | 56[50-62] |  | 61[56-65] |  | 65[60-70] |  | 64[57-71] |  | 67[58-75] |  | 63[56-70] |  |
| No asthma diagnosis | 12[8-19] |  | 25[21-29] |  | 36[30-41] |  | 29[23-36] |  | 36[25-49] |  | 40[29-52] |  | 58[49-67] |  |
|  |  |  |  |  |  |  |  |  |  |  |  |  |  |  |
| Frequent wheeze^#^ | 33[29-38] |  | 57[51-63] |  | 65[60-71] |  | 75[69-80] |  | 71[63-79] |  | 76[64-85] |  | 68[58-77] |  |
| Infrequent wheeze^¶^ | 15[13-18] |  | 29[25-33] |  | 40[35-45] |  | 39[34-44] |  | 41[33-50] |  | 47[38-56] |  | 58[51-65] |  |
|  |  |  |  |  |  |  |  |  |  |  |  |  |  |  |
| *Laughter/crying* |  |  |  |  |  |  |  |  |  |  |  |  |  |  |
| Asthma diagnosis | -* |  | 53[46-60] |  | 46[39-53] |  | 56[49-62] |  | 54[38-69] |  | 42[34-52] |  | 44[38-51] |  |
| No asthma diagnosis | -* |  | 32[26-37] |  | 39[31-47] |  | 27[20-37] |  | 17[3-54] |  | 31[21-42] |  | 37[29-47] |  |
|  |  |  |  |  |  |  |  |  |  |  |  |  |  |  |
| Frequent wheeze^#^ | 49[44-54] |  | 60[52-67] |  | 55[45-64] |  | 70[61-78] |  | 58[37-77] |  | 54[41-66] |  | 52[42-62] |  |
| Infrequent wheeze^¶^ | 29[26-32] |  | 33[28-38] |  | 38[32-44] |  | 36[30-42] |  | 34[19-54] |  | 29[22-38] |  | 37[31-44] |  |
|  |  |  |  |  |  |  |  |  |  |  |  |  |  |  |
| *Dust* |  |  |  |  |  |  |  |  |  |  |  |  |  |  |
| Asthma diagnosis | 7[3-15] |  | 10[5-19] |  | 15[11-20] |  | 18[13-25] |  | 17[12-23] |  | 23[16-31] |  | 39[32-46] |  |
| No asthma diagnosis | 3[1-7] | | 3[1-9] | | 5[3-10] |  | 6[3-14] |  | 12[6-23] |  | 22[14-34] |  | 19[12-28] |  |
|  |  |  |  |  |  |  |  |  |  |  |  |  |  |  |
| Frequent wheeze^#^ | 5[2-11] |  | 10[5-18] |  | 15[11-21] |  | 16[11-24] |  | 23[16-32] |  | 26[16-38] |  | 41[31-51] |  |
| Infrequent wheeze^¶^ | 2.9[1.1-7.4] | | 1.7[1.4-6.8] | | 6[3-10] |  | 12[7-18] |  | 7[4-14] |  | 20[14-29] |  | 27[21-34] |  |
|  |  |  |  |  |  |  |  |  |  |  |  |  |  |  |
| *Pollen* |  |  |  |  |  |  |  |  |  |  |  |  |  |  |
| Asthma diagnosis | - |  | - |  | 33[27-40] |  | 33[27-39] |  | 44[29-60] |  | 42[34-52] |  | 43[37-50] |  |
| No asthma diagnosis | - |  | - |  | 16[11-24] |  | 11[6-19] |  | 17[3-54] |  | 39[28-51] |  | 17[11-26] |  |
|  |  |  |  |  |  |  |  |  |  |  |  |  |  |  |
| Frequent wheeze^#^ | - |  | - |  | 35[26-45] |  | 36[27-45] |  | 38[20-59] |  | 46[34-59] |  | 41[31-51] |  |
| Infrequent wheeze^¶^ | - |  | - |  | 23[18-29] |  | 22[17-28] |  | 38[22-58] |  | 38[30-47] |  | 31[25-38] |  |
|  |  |  |  |  |  |  |  |  |  |  |  |  |  |  |
| *Pets* |  |  |  |  |  |  |  |  |  |  |  |  |  |  |
| Asthma diagnosis | 5[2-12] |  | 12[9-16] |  | 12[9-16] |  | 21[17-25] |  | 17[13-23] |  | 17[11-25] |  | 14[10-20] |  |
| No asthma diagnosis | 2[1-6] |  | 2[1-4] |  | 5[3-8] |  | 5[3-10] |  | 7[3-16] |  | 9[4-19] |  | 6[3-13] |  |
|  |  |  |  |  |  |  |  |  |  |  |  |  |  |  |
| Frequent wheeze^#^ | 8[5-10] |  | 12[8-16] |  | 14[10-18] |  | 25[19-31] |  | 18[12-25] |  | 20[12-32] |  | 13[8-22] |  |
| Infrequent wheeze^¶^ | 1.4[0.8-2.4] | | 2.7[1.5-4.7] | | 6[4-8] |  | 10[7-14] |  | 11[7-18] |  | 10[6-17] |  | 11[7-16] |  |
|  |  |  |  |  |  |  |  |  |  |  |  |  |  |  |
| *Food/drinks* |  |  |  |  |  |  |  |  |  |  |  |  |  |  |
| Asthma diagnosis | 20[13-30] |  | 8[6-12] |  | 12[9-15] |  | 13[10-17] |  | 15[10-21] |  | 16[10-24] |  | 10[7-16] |  |
| No asthma diagnosis | 12[8-18] |  | 11[9-15] |  | 11[8-15] |  | 10[6-15] |  | 16[9-27] |  | 12[6-22] |  | 12[7-20] |  |
|  |  |  |  |  |  |  |  |  |  |  |  |  |  |  |
| Frequent wheeze^#^ | 17[14-20] |  | 11[8-16] |  | 14[11-18] |  | 17[12-22] |  | 17[11-24] |  | 13[7-24] |  | 10[5-18] |  |
| Infrequent wheeze^¶^ | 9[7-11] |  | 9[7-12] |  | 9[7-12] |  | 9[6-12] |  | 14[9-21] |  | 15[9-23] |  | 11[8-17] |  |
|  |  |  |  |  |  |  |  |  |  |  |  |  |  |  |

CI: confidence interval; *: the questions on doctor-diagnosed asthma and cough asked in different subcohorts in 1998; ^#^: ≥4 attacks of wheeze in the past 12 months; ^¶^: <4 attacks of wheeze in the past 12 months.
