# Supplementary material for: Prevalence of cough throughout childhood: A cohort study
Source: PLoS One. 2017 May 24;12(5):e0177485. doi: 10.1371/journal.pone.0177485 (PMC5443519; doi:10.1371/journal.pone.0177485)
Supplement: S8 Table — (DOCX) [file pone.0177485.s012.docx]

**S8 Table. Unweighted and weighted prevalence of cough in the entire cohort of children, children with wheeze and children without wheeze.**

| **Age group** | **1-year-olds**  **(N=4102)** | | **2-year-olds**  **(N=3163)** | | **3-4-year-olds**  **(N=4071)** | | **5-6-year-olds**  **(N=4031)** | | **7-9-year-olds**  **(N=3244)** | | **10-13-year-olds (N=2204)** | | **14-17-year-olds (N=2025)** | |
| --- | --- | --- | --- | --- | --- | --- | --- | --- | --- | --- | --- | --- | --- | --- |
| *Entire cohort* | %[CI] | w.%[CI] | %[CI] | w.%[CI] | %[CI] | w.%[CI] | %[CI] | w.%[CI] | %[CI] | w.%[CI] | %[CI] | w.%[CI] | %[CI] | w.%[CI] |
| Coughing more^#^ | 10[9-11] | 10[8-11] | 9[8-10] | 10[8-11] | 11[10-13] | 12[10-13] | 11[10-12] | 12[10-13] | 10[9-12] | 11[9-13] | 10[9-12] | 11[10-13] | 11[10-12] | 11[10-13] |
| Cough with colds | 68[66-69] | 69[67-70] | 70[68-71] | 71[69-73] | 75[73-76] | 75[74-77] | 71[70-73] | 72[70-73] | 64[63-66] | 66[64-68] | 67[65-69] | 67[64-69] | 68[66-70] | 69[67-71] |
| Cough without colds | 34[33-36] | 33[31-35] | 37[35-38] | 36[34-38] | 38[36-39] | 38[36-40] | 39[38-41] | 39[38-41] | 36[35-38] | 35[33-37] | 47[45-49] | 49[46-51] | 55[52-57] | 56[53-58] |
| Night cough^¶^ | 23[21-24] | 23[21-24] | 24[22-25] | 25[23-27] | 31[29-32] | 32[30-33] | 27[26-28] | 28[26-29] | 25[24-27] | 26[25-28] | 22[20-24] | 23[21-25] | 20[19-22] | 21[19-23] |
| *Cough triggers*^¶^*:* |  |  |  |  |  |  |  |  |  |  |  |  |  |  |
| Exercise/play^#^ | 10[9-11] | 10[9-11] | 16[15-17] | 18[16-19] | 20[19-22] | 21[20-23] | 18[17-19] | 19[18-20] | 16[15-18] | 17[15-19] | 25[22-27] | 26[23-30] | 26[24-28] | 28[26-30] |
| Laughter/crying^#^ | 22[21-24] | 22[21-24] | 22[20-24] | 23[21-25] | 22[21-24] | 24[22-26] | 19[18-21] | 20[18-22] | 22[16-28] | 23[17-30] | 18[16-21] | 21[18-24] | 26[24-28] | 27[25-30] |
| Dust^#^ | 2[1-3] | 2[1-4] | 2[1-3] | 2[1-4] | 4[3-5] | 4[3-5] | 4[3-5] | 4[3-5] | 5[4-6] | 5[4-6] | 9[7-11] | 10[8-13] | 14[12-15] | 14[12-15] |
| Pollen^#^ | - | - | - | - | 10[8-11] | 10[9-12] | 8[7-9] | 8[7-10] | 15[11-21] | 16[11-22] | 17[14-19] | 17[15-20] | 16[15-18] | 16[15-18] |
| Pets^#^ | 2[1-2] | 1[1-2] | 2[1-2] | 2[1-2] | 2[2-3] | 2[2-3 | 3[3-4] | 3[3-4] | 3[3-4] | 3[3-4] | 4[3-5] | 4[3-6] | 3[3-4] | 3[3-4] |
| Food/drinks^#^ | 11[10-12] | 11[10-12] | 9[8-10] | 9[8-11] | 8[8-9] | 8[7-9] | 7[6-7] | 6[6-7] | 6[5-7] | 6[5-7] | 6[5-8] | 7[6-10] | 7[6-8] | 7[6-9] |
|  |  |  |  |  |  |  |  |  |  |  |  |  |  |  |
|  | **1-year-olds**  **(N=1409)** | | **2-year-olds**  **(N=726)** | | **3-4-year-olds**  **(N=761)** | | **5-6-year-olds**  **(N=606)** | | **7-9-year-olds**  **(N=434)** | | **10-13-year-olds (N=331)** | | **14-17-year-olds**  **(N= 309)** | |
| *Wheezers* | %[CI] | w.%[CI] | %[CI] | w.%[CI] | %[CI] | w.%[CI] | %[CI] | w.%[CI] | %[CI] | w.%[CI] | %[CI] | w.%[CI] | %[CI] | w.%[CI] |
| Coughing more^#^ | 22[19-24] | 21[18-24] | 27[24-31] | 30[25-34] | 35[30-40] | 35[30-41] | 42[37-47] | 43[38-49] | 34[28-41] | 39[31-47] | 38[33-44] | 42[36-49] | 34[29-39] | 34[29-41] |
| Cough with colds | 84[82-86] | 85[82-87] | 88[86-90] | 89[86-91] | 91[89-93] | 91[88-93] | 91[88-93] | 90[88-93] | 87[84-90] | 88[85-91] | 87[83-91] | 87[83-91] | 85[81-89] | 85[80-89] |
| Cough without colds | 51[48-54] | 47[44-51] | 56[53-60] | 56[51-60] | 62[59-66] | 63[58-67] | 66[62-69] | 65[61-69] | 68[64-73] | 69[65-73] | 75[70-79] | 76[70-81] | 74[69-79] | 74[68-79] |
| Night cough^¶^ | 36[33-38] | 35[31-38] | 44[41-48] | 45[41-50] | 55[52-59] | 55[51-59] | 53[49-57] | 53[49-57] | 57[52-62] | 60[55-64] | 52[47-57] | 55[49-61] | 40[34-45] | 39[33-45] |
| *Cough triggers*^¶^*:* |  |  |  |  |  |  |  |  |  |  |  |  |  |  |
| Exercise/play^#^ | 22[20-24] | 21[19-24] | 39[35-42] | 41[36-45] | 50[47-54] | 51[47-55] | 53[49-57] | 55[50-59] | 56[50-62] | 59[52-65] | 57[50-64] | 58[50-66] | 61[56-67] | 64[58-70] |
| Laughter/crying^#^ | 36[33-39] | 35[32-39] | 41[37-46] | 41[36-46] | 43[38-49] | 44[38-50] | 47[42-53] | 48[42-53] | 45[32-59] | 47[33-61] | 38[31-45] | 42[34-50] | 42[36-48] | 42[36-49] |
| Dust^#^ | 4[2-7] | 5[2-11] | 5[3-9] | 6[2-14] | 10[8-14] | 13[9-18] | 14[10-19] | 15[10-22] | 15[11-20] | 16[12-21] | 22[17-29] | 25[18-32] | 32[27-37] | 30[25-37] |
| Pollen^#^ | - | - | - | - | 27[22-32] | 26[21-32] | 26[22-31] | 27[22-32] | 38[25-52] | 40[27-55] | 41[34-48] | 42[35-50] | 34[29-40] | 35[29-41] |
| Pets^#^ | 4[3-5] | 3[2-4] | 6[4-8] | 5[4-8] | 9[7-11] | 8[6-11] | 16[13-19] | 16[13-20] | 14[11-19] | 14[10-19] | 14[9-20] | 14[10-21] | 12[8-16] | 12[8-17] |
| Food/drinks^#^ | 12[10-14] | 12[10-14] | 10[8-12] | 11[8-15] | 11[9-14] | 12[9-15] | 12[10-15] | 12[9-15] | 15[11-20] | 16[12-20] | 14[10-20] | 13[9-19] | 11[8-15] | 10[7-15] |
|  |  |  |  |  |  |  |  |  |  |  |  |  |  |  |
|  | **1-year-olds**  **(N=2693)** | | **2-year-olds**  **(N=2437)** | | **3-4-year-olds**  **(N=3310)** | | **5-6-year-olds**  **(N=3425)** | | **7-9-year-olds**  **(N=2810)** | | **10-13-year-olds (N=1873)** | | **14-17-year-olds (N=1716)** | |
| *Non-wheezers* | %[CI] | w.%[CI] | %[CI] | w.%[CI] | %[CI] | w.%[CI] | %[CI] | w.%[CI] | %[CI] | w.%[CI] | %[CI] | w.%[CI] | %[CI] | w.%[CI] |
| Coughing more^#^ | 3[2-4] | 3[2-4] | 4[3-5] | 4[3-5] | 6[5-8] | 6[5-8] | 6[5-7] | 6[5-7] | 6[5-7] | 5[4-7] | 5[4-6] | 6[5-7] | 7[6-8] | 7[6-9] |
| Cough with colds | 60[58-62] | 60[58-63] | 64[62-66] | 66[63-68] | 71[69-73] | 72[70-73] | 68[66-70] | 68[67-70] | 61[59-63] | 63[61-64] | 64[61-66] | 63[60-66] | 65[63-67] | 66[63-68] |
| Cough without colds | 25[24-27] | 25[23-27] | 31[29-33] | 30[28-32] | 32[31-34] | 32[30-33] | 34[33-36] | 34[33-36] | 31[30-33] | 30[28-32] | 42[40-44] | 44[42-47] | 51[49-53] | 52[49-55] |
| Night cough^¶^ | 16[14-17] | 16[15-18] | 18[16-19] | 19[17-21] | 25[23-26] | 26[24-28] | 22[21-24] | 23[21-24] | 21[19-22] | 21[19-22] | 17[15-19] | 18[15-20] | 17[15-19] | 18[16-20] |
| *Cough triggers*^¶^*:* |  |  |  |  |  |  |  |  |  |  |  |  |  |  |
| Exercise/play^#^ | 5[4-5] | 4[3-5] | 10[8-11] | 11[9-12] | 14[12-15] | 14[13-16] | 12[11-13] | 12[11-14] | 10[9-12] | 11[9-12] | 17[14-20] | 19[16-22] | 20[18-22] | 21[19-24] |
| Laughter/crying^#^ | 15[13-17] | 15[13-17] | 16[15-18] | 18[16-20] | 18[16-20] | 19[17-21] | 14[13-16] | 14[13-16] | 13[8-19] | 12[7-19] | 14[12-16] | 16[13-19] | 23[21-25] | 25[22-27] |
| Dust^#^ | 1.4[0.7-2.9] | 1.0[0.5-2.3] | 0.5[0.2-1.5] | 0.5[0.1-1.6] | 2[2-3] | 2[1-2] | 2[1-3] | 2[1-3] | 3[2-4] | 3[3-4] | 6[4-7] | 7[5-9] | 11[9-12] | 11[9-12] |
| Pollen^#^ | - | - | - | - | 6[5-7] | 7[5-8] | 5[4-6] | 5[4-6] | 7[3-12] | 5[3-11] | 11[9-13] | 11[9-14] | 13[11-15] | 13[11-15] |
| Pets^#^ | 0.5[0.3-0.9] | 0.5[0.2-1.0] | 0.6[0.4-1.0] | 0.6[0.3-1.0] | 0.9[0.6-1.3] | 0.8[0.5-1.1] | 1.2[0.9-1.7] | 1.2[0.9-1.6] | 1.5[1.0-2.2] | 1.6[1.1-2.3] | 1.7[1.0-2.9] | 2.0[1.1-3.6] | 1.9[1.4-2.7] | 1.9[1.3-2.8] |
| Food/drinks^#^ | 10[9-11] | 10[9-12] | 9[8-10] | 9[8-10] | 8[7-9] | 8[7-9] | 6[5-7] | 5[5-6] | 4[3-5] | 5[4-6] | 4[3-6] | 6[4-9] | 6[5-7] | 7[5-8] |
|  |  |  |  |  |  |  |  |  |  |  |  |  |  |  |

%[CI]: unweighted prevalence with confidence intervals;

w.%[CI]: weighted prevalence with confidence intervals;

^#^: only asked in part of the cohort;

^¶^: symptoms occurring in the past 12 months.
